# The Role of microRNAs, Long Non-coding RNAs, and Circular RNAs in Cervical Cancer

**DOI:** 10.3389/fonc.2020.00150

**Published:** 2020-02-20

**Authors:** Maria Lina Tornesello, Raffaella Faraonio, Luigi Buonaguro, Clorinda Annunziata, Noemy Starita, Andrea Cerasuolo, Francesca Pezzuto, Anna Lucia Tornesello, Franco Maria Buonaguro

**Affiliations:** ^1^Molecular Biology and Viral Oncology Unit, Istituto Nazionale Tumori IRCCS “Fondazione G. Pascale”, Naples, Italy; ^2^Department of Molecular Medicine and Medical Biotechnology, University of Naples Federico II, Naples, Italy; ^3^Cancer Immunoregulation Unit, Istituto Nazionale Tumori IRCCS “Fondazione G. Pascale”, Naples, Italy

**Keywords:** cervical cancer, long non-coding RNA, circular RNA, microRNA, human papillomavirus (HPV)

## Abstract

Prolonged infection of uterine cervix epithelium with human papillomavirus (HPV) and constitutive expression of viral oncogenes have been recognized as the main cause of the complex molecular changes leading to transformation of cervical epithelial cells. Deregulated expression of microRNAs (miRNA), long non-coding RNAs (lncRNA), and circular RNAs (circRNA) is involved in the initiation and promotion processes of cervical cancer development. Expression profiling of small RNAs in cervical neoplasia revealed up-regulated “oncogenic” miRNAs, such as miR-10a, miR-21, miR-19, and miR-146a, and down regulated “tumor suppressive” miRNAs, including miR-29a, miR-372, miR-214, and miR-218, associated with cell growth, malignant transformation, cell migration, and invasion. Also several lncRNAs, comprising among others HOTAIR, MALAT1, GAS5, and MEG3, have shown to be associated with various pathogenic processes such as tumor progression, invasion as well as therapeutic resistance and emerged as new diagnostic and prognostic biomarkers in cervical cancer. Moreover, human genes encoded circular RNAs, such as has_circ-0018289, have shown to sponge specific miRNAs and to concur to the deregulation of target genes. Viral encoded circE7 has also demonstrated to overexpress E7 oncoprotein thus contributing to cell transformation. In this review, we summarize current literature on the complex interplay between miRNAs, lncRNAs, and circRNAs and their role in cervical neoplasia.

## Introduction

Cervical cancer is the fourth most frequently diagnosed tumor and the fourth leading cause of cancer death in women in the world with ~570,000 cases and 311,000 deaths in 2018 (1). The persistent infection with carcinogenic human papillomaviruses (HPV) has shown to be the necessary cause of ~95% of invasive cervical cancer, including cervical squamous cell carcinoma (SCC) and adenocarcinoma (AC) histotypes (2). Cervical SCC is generally preceded by persistent squamous intraepithelial lesions (SIL) caused by HPV infection, therefore the detection of viral nucleic acids has shown to be valuable for the effective prevention of cervical cancer development in oncologic screening programs (3).

The E6 and E7 oncoproteins encoded by high risk HPVs are considered the main players of the multistep transformation process affecting the infected cervical cells. Indeed, they are able to inhibit p53 and pRb oncosuppressors, respectively, and to interact with a plethora of cell signaling factors regulating cell cycle, genome stability and epigenetic modifications (4, 5). Moreover, the HPV E5 protein has also a relevant role in tumor cell invasion and metastasis for its ability to increase the expression of the epidermal growth factor receptor (EGFR) and c-MET, the latter being also critical for viral gene expression (6, 7).

Nevertheless, the gradually accumulation of genetic and epigenetic alterations in HPV infected cells is also crucial for the ultimate progression to cervical cancer. The mutational profile of cervical carcinoma showed the presence of non-synonymous somatic nucleotide changes in PIK3CA, PTEN, TP53, STK11, and KRAS genes (8–11). Recent advances in cancer genome sequencing allowed to identify further unknown mutations in MAPK1, HLA, EP300, FBXW7, NFE2L2, ERBB3, CASP8, TGFBR2, and SHKBP1 genes as well as sequence amplifications in CD274 (PD-L1), PDCD1LG2 (PD-L2), and BCAR4 (lncRNA BCAR4) genes (12, 13). In addition, activating mutations creating *de novo* transcription factor binding sites in regulatory regions, such as the TERT promoter sequence, have been identified in a significant fraction of cervical SCC (14).

**Figure 1:**
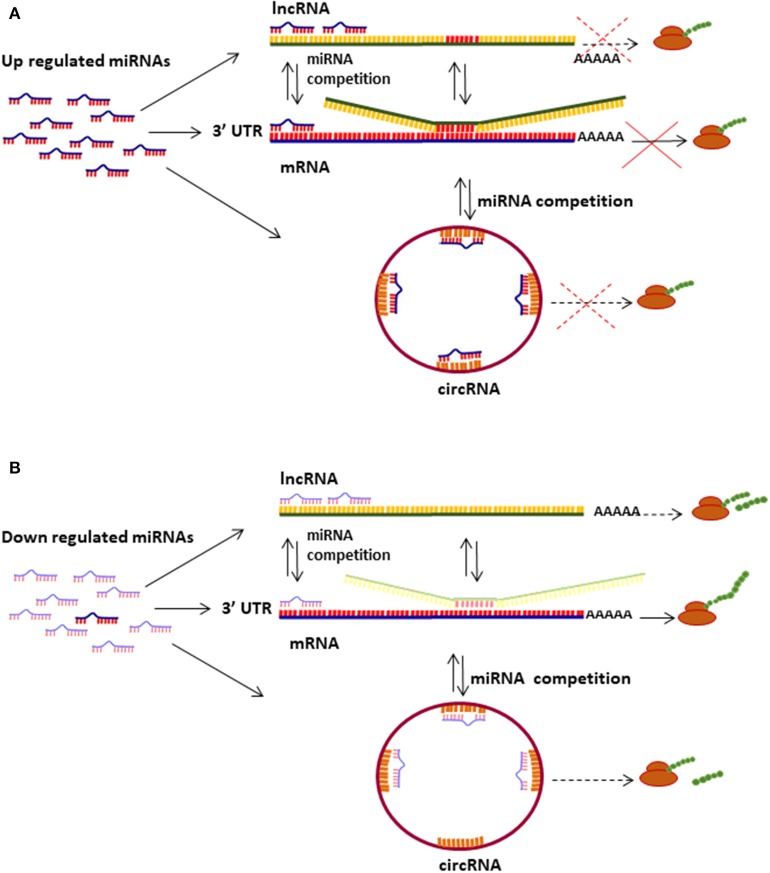
Mechanisms of miRNas/lncRNAs/circRNAs regulating mRNA translation. **(A)** Up regulated miRNAs bind to mRNA 3′UTR and inhibit translation depending on the sponging effect of lncRNAs and circRNAs. **(B)** miRNAs down regulation induces protein translation from diverse types of transcripts (mRNA, lncRNA, and circRNA); several lncRNAs and circRNAs can be translated into small proteins and peptides.

Epigenetic modifications, including deregulation of microRNA (miRNA), long non-protein coding RNA (lncRNA) and circular RNA (circRNA) levels, have shown to play important roles in cell transformation during distinct stages of cervical intraepithelial neoplasia and cervical carcinoma development [Figure 1; (15–17)].

MiRNAs are small (19–25 nucleotides long), single-stranded non-coding RNAs that regulate gene expression mainly by binding to sequence motifs located within the 3′ untranslated region (UTR) of mRNA transcripts (18, 19). Other regulatory functions include their reciprocal interaction on primary miRNA transcription processes, binding to double-stranded DNA to form triple helixes as well as interaction with RNA G-quadruplex structures that interfere at specific gene regulatory sites (20). The differential expression of the ~2,500 miRNAs encoded by the human genome has an important role in the embryo development and in the physiological functioning of tissues and organs (21, 22). Several miRNAs have oncogenic or tumor suppressor activities and play a fundamental role in cancer development, progression and dissemination (23). A recent meta-analysis of miRNA profiles in cervical neoplasia cases and normal cervical epithelium samples identified 42 up regulated and 21 down regulated miRNAs among different stages of cervical neoplasia (24). The pathway enrichment analysis of genes targeted by these miRNAs revealed the alteration of p53, ErbB, MAPK, mTOR, Notch, TGFβ, and Wnt pathways all contributing to hallmarks of cancer (24).

lncRNAs are regulatory transcripts longer than 200 nucleotides mostly transcribed by RNA pol II and characterized by a 5′ 7-methylguanosine cap and a 3′ poly (A) tail similarly to messenger RNAs (25). Despite being not translated into full-length proteins, lncRNAs are implicated in a variety of biological activities such as regulation of gene transcription mediated by their interaction with chromatin-modifying complexes at specific regulatory regions, decoy for transcription factors and miRNAs as well as scaffolding for functional ribonucleoprotein complexes organization (26, 27). Deregulation of lncRNAs expression is associated with cardiovascular and neurodegenerative diseases as well as with cancer development (27, 28). Around 14 lncRNAs have shown to be altered in cervical carcinoma affecting important metabolic pathways such as STAT3, wnt/β-catenin, PI3K/AKT, and Notch signaling (29). Moreover, some lncRNAs, including MALAT1, CCEPR, and TMPOP2, are reciprocally regulated by HPV16 E6 and E7 expression hence enhancing the oncogenic effect of viral oncoproteins in the progression of cervical neoplasia (30, 31).

The single-stranded closed RNA molecules (circRNA) are a new class of non-coding RNAs, originating from back-splicing of pre-mRNAs, that have several biological functions in normal cells including the ability to act as sponges to efficiently subtract microRNAs and proteins (32). CircRNAs have shown to be aberrantly expressed in a tissue-specific manner in cancer cells and to contribute to cancer development by perturbing cell proliferation, migration, and angiogenesis processes (33). Several studies indicated that circRNAs play a significant role in cervical cancer development by different molecular mechanisms, which among them miRNA sponging is the most important (34). A recent study investigating circRNA expression in cervical cancer tissues by microarray analysis showed that 45 circRNAs were upregulated and that the most expressed has_circ_0018289 was involved in the direct binding of miR-497 (35).

The aim of this review is to summarize the recent studies on the role of miRNAs, lncRNAs, and circRNAs as well as their reciprocal regulation in different stages of cervical neoplasia. Moreover, it provides an overview of the potential impact of non-coding RNAs in the diagnosis and therapy of cervical cancer.

## The Role of miRNAs in Cervical Neoplasia

Many studies have evaluated the expression levels of miRNAs in cervical neoplasia biopsies as well as in exfoliated cervical cells, in cervical mucus and in the serum of women diagnosed with cervical cancer (Table 1). The first study describing the differential expression of six miRNAs (let-7b, let-7c, miR-21, miR-23b, miR-196b, and miR-143) in human cervical carcinoma cell lines in comparison with normal cervical samples was published by Lui et al. (107). Numerous investigations since then have been conducted in order to characterize the mechanisms causing miRNAs deregulation as well as their expression pattern in cervical cancer tissues vs. normal cervical epithelia. Indeed, genetic alterations of miRNA loci such as gene deletions, amplifications, or mutations as well as epigenetic silencing such as DNA methylation or deregulation of miRNA processors and transcription factors have been all associated with aberrant expression of miRNAs in cervical cancer (108). For example, Wilting et al. identified 89 deregulated miRNAs in cervical SCC with miR-9 over expression significantly associated with chromosome 1q gain and with increased cell viability, anchorage-independent growth and cell migration (36). Moreover, Muralidhar et al. identified 16 deregulated miRNAs, including miR-21, miR-29a, miR-31, and miR-203, in advanced cervical SCC which were associated with the up regulation of miRNA processor Drosha transcript and gain of chromosome 5p (109).

**Table 1:** miRNAs identified in cervical tissues, cell exfoliates, and mucus as well as in the serum of high grade SIL and cervical cancer patients.

Deregulated miRNAs^*^	HSIL^**^	**Cervical Cancer**	**References**
Up regulated miRNAs in tissue biopsies		*miR-20a*-*5p*; *miR-31-5p*; ***miR-96-5p***; *miR-142-5p*; *miR-18*9-5p; miR-20*0b*; *miR-224-5p*; *miR-944*; miR-*1246*; *let*-*5p*;	(24, 36–62)
	let-7; *miR-10a-5p*; *miR-16-5p*; miR21-5p; *miR-25-5p*; *miR-26a; miR-29a; miR-29b; miR-29c; miR-30a; miR-34b; miR-34c-5p*; *miR-92a-3p*; *miR-101; miR-125a-5p; miR-135b; miR- 143; miR-145*; *miR-196a-5p*; *miR-223; miR-338; miR-301b; miR-345; miR-424; miR-466; miR-512-5p; miR-518a*	***miR-9-5p***; ***miR-10a-5p***; **miR-15b**; ***miR-16-5p***; **miR-17**; miRNA-19a/b; **miR-20b**; **miR-21-5p**; ***miR-25-5p***; **miR-27a**; miR-29a; miR-30a; ***miR-92a-3p***; **miR-92b**; **miR-93**; **miR-106a**; miR-125b; miR-127; miR-130a; miR-133a; miR-133b; miR-135b; miR-141b; miR-145b; **miR-146a**; miR-150; **miR-155**; miR-181b; miR-182; **miR-185**; *miR-196a-5p*; miR-199a; **miR-199a**; miR-199b; miR-199-s; miR-203b; miR-205; miR-215; miR-221; miR-222; miR-223; miR-301b; miR-320a; miR-361-5p; miR-373; miR-378; miR-425-5p; miR-449; miR-451a; miR-466; miR-486-5p miR-494; miR-500; miR-505; miR-519d; miR-543; miR-590-5p; miR-711; miR-720; miR-886-5p; miR-888; miR-892b; miR-944; miR-1290; miR-2392; miR-3147; miR-3162; miR-4484; miR-6852;	
Down regulated miRNAs in tissue biopsies		*miR-1*; *miR-99b*-*5p*; *miR-126-3p*; *miR-140-5p*; *miR-196b*-*5p*;	(24, 36, 37, 39, 40, 55, 58, 63–93)
	Let-7a; miR-22; *miR-29a*; *miR-34a; miR-99a-5p; miR-100-5p*; miR-129b-5p; miR-193a-3p; *miR-199a-3p*; *miR-203*; miR-205; miR-216-5p; *miR-218-5p*; miR-212; miR-221; miR-27a; miR-27b; miR-342; miR-376c-3p^***^; miR-433; miR-484; miR-636; miR-770-5p	Let-7a; Let-7b; Let-7c; Let-7g; miR-7; miR-10b; miR-17-5p; miR-22; miR-24; *miR-26a*; miR-27b; ***miR-29a***; miR-29b; miR-30a; miR-30e; ***miR-34a***; ***miR-99a-5p***; ***miR-100*****-*****5p***; miR-101; miR-103b; miR-107; miR-124-3p; miR-125a-5p; **miR-125b**; miR-129b-5p; miR-132; miR-133a; miR-138; miR-139-3p; miR-141; miR-142-3p; miR-143; miR-144; **miR-145**; miR-149; miR-152; miR-154; miR-181; miR-182; miR-183; miR-186; miR-187; miR-193a; **miR-193b; miR-195**; miR-199a-3p; miR-199b; miR-200b; miR-200c; miR-202; ***miR-203***; miR-204; miR-205; miR-211; miR-212; miR-214; miR-216-5p; ***miR-218***; miR-223; miR-296; miR-320; miR-326; miR-328; miR-329; miR-331-3p; miR-335; miR-337; miR-338-3p; miR-342; miR-362; miR-374c-5p; **miR-375**; miR-376c; miR-379; miR-383; miR-376a; **miR-424**; miR-429; miR-451; miR-484; miR-486-3p; miR-489-3p; miR-491-5p; miR-494; **miR-497**; miR-503; miR-506; miR-544; miR-630; miR-634; miR-638; miR-720; miR-758; miR-892b; miR-1297; miR-1246; miR-2861; miR-3185; miR- 3156-3p; miR- 3666; miR-3960; miR-4262; miR-4467; miR-4488; miR-4525	
Up regulated miRNAs in cervical exfoliated cells	miRNA-16-2; miRNA-20a		(94)
Down regulated miRNAs in exfoliated cervical cells	miR-424; miR-375; *miR-34a*; miR-218; miRNA-195; miRNA-29a	miR-758	(93–95)
Up regulated miRNAs in the serum		*miR-20a-5p*	(39, 59, 61, 96–103)
	miR-9-5p; *miR-10a-5p*; miR-20a-5p; *miR-92a-3p*; miR-196a-5p;	**miR-9-5p**; **miR-21-5p**; miR-29a-3p; *miR-92a-3p*; miR-101-3p; miR-122-5p; miR-132-3p; miR-141-3p; miR-150; **miR-155**; miR-191-5p; *miR-196a-5p*; miR-200c-3p; miR-203a-3p; miR-205-5p; miR-212-3p; miR-214–3p; miR-370; miR-425-5p; miR-486-5p; miR-494; miR-1246	
Down regulated miRNAs in the serum	Let-7a-5p; miR-101; miR-142-3p	Let-7a-5p; miR-17-5p; miR-24; miR-101; miR-103a-3p; miR-106a-5p; miR-106a-5p; miR-139-3p; miR-142-3p miR-144-3p; miR-191-5p**; miR-195-5p**; miR-212-3p; ***miR-218-5p***; miR-370; miR-425-5p; miR-451a; miR-758	(93, 99, 102, 104–106)
Up regulated miRNAs in cervical mucus	Let-7a-5p; *miR-10a-5p*; miR-21-5p; miR-141-3p; miR-144-3p; miR-155-5p; miR-205-5p; miR-451a	Let-7a-5p; ***miR-10a-5p***; **miR-17-5p**; **miR-21-5p**; **miR-106a-5p**; miR-141-3p; miR-144-3p; **miR-155-5p**; miR-205-5p; miR-451a; miR-758;	(96)
Down regulated miRNAs in cervical mucus	Not reported	Not reported	

* *miRNAs up-regulated or down regulated in more than one study as determined by Pardini et al. (37) have been highlighted in bold; miRNAs with statistically significant expression changes in cervical neoplasia samples vs. normal epithelia as reported by He et al. (24) have been written in italics; miRNAs which have been found specifically expressed in in cervical cancer and not in HSIL are underlined and listed as a separated group*.

** *HSIL group comprises CIN2-3 lesions*.

*** *The miR-376c-3p has been found significantly down regulated in CIN3 compared to CIN2 (24)*.

Numerous studies over the past decade have analyzed the miRNAs expression to identify significant variations during the transition from low to high grade cervical neoplasia and to invasive cervical cancer in order to define novel biomarkers for cervical cancer diagnosis, prognosis and cancer stage (24, 37). Pereira et al. described eight down regulated miRNAs (miR-26a, miR-29a, miR-99a, miR-143, miR-145, miR-199a, miR-203, miR-513) and five upregulated miRNAs (miR-148a, miR-302b, miR-10a, miR-196a, and miR-132) in low SIL, high SIL and cervical SCC in comparison with normal samples (110). Only five miRNAs (miR-106a, miR-197, miR-16, miR-27a, and miR-142-5p) were found specifically deregulated in pre-neoplastic lesions but not in cervical carcinoma (110). Subsequently, many research groups reported the identification of specific miRNA signatures during the transition from SIL to cervical cancer with variable results mainly due to the small sample size, the type of specimens (i.e., formalin fixed paraffin embedded vs. fresh biopsies), the number of miRNAs included in each panel (ranging from one to 7788 miRNAs) as well as the diverse methods used to quantify (37, 96, 111, 112).

He et al. performed a systematic study to identify miRNAs with statistically significant expression changes among 827 CIN and 3,095 cervical carcinomas as well as 2,099 non-tumor tissues from 85 selected studies (24). All studies analyzing miRNA profile in human cancerous and non-cancerous cervical tissues were included in such analysis. This approach allowed to identify 42 up regulated and 21 down-regulated miRNAs in different stages of cervical neoplasia. In particular, seven miRNAs (miR-29a, miR-34a, miR-99a-5p, miR-100-5p, miR-199a-3p, miR-203, and miR-218-5p) were found down-regulated and five miRNAs (miR10a-5p, 16-5p, 25-5p, 92a-3p, and 196a-5p) up regulated in CIN1 as well as in CIN 2–3 and cervical SCC compared to cervical non-tumor tissues. In CIN2-3 there were eight additional down regulated miRNAs and 27 new upregulated miRNAs with the miR-376c-3p specifically down regulated in CIN3 compared to CIN2. The functional target of miR-376c-3p is the BMI1 polycomb ring finger proto-oncogene which is highly expressed in several cancer types, including cervical cancer (113). Thus, the miR-376c-3p and the BMI1 factor may represent novel biomarkers for diagnosis as well as for target therapy in cervical cancer patients. Moreover, five new down regulated (miR-1, miR-99b-5p, miR-126-3p, miR-140-5p, miR-196b-5p) and ten additional up-regulated miRNAs (miR-20a-5p, miR-31-5p, miR-96-5p, miR-142-5p, miR-189-5p, miR224-5p, miR-200b, miR-944, miR-1246, and Let-5p) were identified in cervical carcinoma compared to CIN3 miRNA profile (24). Among these, miR-96-5p and miR-126-3p, which are predicted to target PTEN and MARK1 tumor-associated genes, have shown to be associated with metastatic potential of cervical cancer and to have a great potential as prognostic biomarkers or therapeutic targets (114, 115).

The expression levels of miRNAs consistently altered in all stages of cervical neoplasia may be directly deregulated by early viral proteins produced soon after infection. Gocze et al. performed the miRNA profiling of cervical neoplasia biopsies and identified the over expression of miR-21, miR-34a, miR-196a, miR-27a, and miR-221 as specific signature of HPV positivity irrespective of the clinical tumor grading (38). Indeed, the E6 protein encoded by oncogenic HPVs has shown to down regulate both the tumor suppressors miR-34a, through the viral E6-mediated degradation of the oncosuppressor p53 (116, 117), and the miR-218, in a p53-independent manner thus causing over expression of its target laminin 5 β3 (LAMB3) gene in cervical SCC cells (63, 118). Furthermore, the HPV E6 was demonstrated to up regulate miR-20a thereby enhancing cell proliferation, through the AKT/p38 pathway activation, and tumor growth by down regulation of the target gene PDCD6 (119). The E6 protein has also shown to increase miR-20b levels which in turn inhibits metastasis suppressor TIMP-2 expression and promotes epithelial-mesenchymal transition, migration and invasion of cervical cancer cells (120). Both E6 and E7 oncoproteins have shown to strongly suppress miR-424 levels in HPV16 and HPV31-positive cells causing increased expression of its target gene CHK1 encoding a damage repair factor (25).

Integration of HPV DNA into host genome, with disruption of E2 viral gene and host chromosomal loci, is a critical event in cancer development and progression. Interestingly, the viral status has shown to affect the levels of several miRNAs. Mandal et al. reported that cervical cancer biopsies either carrying integrated or episomal HPV16 genomes have a common expression signature of miR-200a associated with HPV16 genotype. Conversely, down regulation of miR-181c and expression of its target gene CKS1B was observed only in cases harboring HPV16 episomes but not in cancer tissues with the integrated virus (121). Therefore, miR-200a and miR-181c could represent useful biomarkers of cervical neoplasia progression if will be validated in clinical studies.

The HPV genome encodes the HPV-16-miR-H1-1 and HPV-16-miR-H2-1 which, besides being essential for viral infection and maintenance, are able to target critical cell genes, including those regulating cell cycle progression, migration, and immunological response (122). On the other hand, the miR-375 has shown to suppress HPV E6 and E7 expression and to induce increased levels of p53 and p21, higher activity of caspase-3 and caspase-9, suppression of E6AP, IGF-1R, cyclin D1, and survivin protein expression (123). Development of therapeutic strategies able to increase miR-375 levels and re-expression of tumor suppressors may be effective to improve the clinical outcomes in cervical cancer patients.

Recently, Pardini et al. performed a comprehensive evaluation of those miRNAs found deregulated in more than one study in order to identify consistent signatures occurring during the progression from normal cervical epithelium to SIL stages and cervical SCC. Among the 24 studies included in the analysis 17 and 13 miRNAs were found up or down regulated, respectively, in relation to cervical carcinoma progression (37). Among the over expressed miRNAs, one (miR-21) was found associated with cervical carcinogenesis in five studies, and nine (miR-9, miR-16, miR-25, miR-10a, miR-20b, miR-31 miR-92a, miR-106a, and miR-155) in three studies. Among under expressed miRNAs, miR-218 was described in six studies, miR-375 and miR-203 in four studies and six miRNAs (miR-99a, miR-29a, miR-195, miR-125b, miR-34a, and miR-100) in three studies. These findings suggest that specific panels including commonly deregulated miRNAs may serve as effective diagnostic biomarkers for early diagnosis and disease progression as well as for therapy in cervical cancer patients.

Identification of novel cancer biomarkers in cervical exfoliated cells as well as biological fluids may have an important role in early cancer detection and/or recurrence monitoring following therapy. Interestingly, Tian et al. showed that the expression level of miR-424, miR-375, miR-34a, and miR-218 in cervical exfoliated cells was statistically significant lower in high grade lesions than in low-grade lesions with a sensitivity superior to the cytology, suggesting that their detection may provide a new triage choice for the follow up of HPV-positive women (95). Moreover, the levels of miR-34a, miR-125, and miR-375 were also found deregulated in cervical exfoliated cells in association with cancer progression (37). Accordingly, Ye et al. observed that the relative low expression of miRNA-195 and miRNA-29a and the high expression of miRNA16-2 and miRNA20a in the cervical exfoliated cells was predictive of high grade SIL in the women group diagnosed with low grade SIL. Among these, miR-29a expression achieved the highest sensitivity (92.6%) and specificity (80.7%) in the identification of high grade SIL (94). These results indicate that miRNA expression profiles may represent promising biomarkers for the early diagnosis of high grade cervical lesions and cervical cancer.

Profiling of miRNAs in cervical mucus has also shown to be a good strategy for the identification of cervical neoplastic lesions (96). Indeed, four miRNAs (miR-126-3p, miR-20b-5p, miR-451a, and miR-144-3p) detected in cervical mucus have been shown effective for diagnosis of cervical adenocarcinoma and high-grade intraepithelial lesions. However, more investigations are needed in order to establish the clinical value of miRNA detection in cervical mucus.

Few studies evaluated the parallel expression of miRNAs in cervical cancer biopsies and in serum samples and the potential of circulating miRNAs as cancer biomarkers. Chen et al. examined the levels of 1,450 miRNAs in cervical carcinoma tissues as well as in sera and identified 62 up regulated and 27 down regulated in comparison with normal controls (39). Among these, the miR-1246, miR-20a, miR-2392, miR-3147, miR-3162-5p, miR-4484, and miR-4667-5p were all over expressed in patients sera consistently with their high levels in cervical cancer tissues (39). More recently, Shukla et al. identified 14 miRNAs differentially expressed in cervical cancer tissue and serum specimens by performing sequencing analysis and real time PCR (124). Among them, miR-17-5p, miR-32-5p, and miR-454-3p were over expressed while miR-409-3p was down regulated both in serum samples and tumor biopsies of cervical cancer patients. Moreover, an inverse correlation was observed between the miR-409-3p and miR-454-3p levels and the expression of their target genes MTF2 and ST18, respectively, in cervical cancer biopsies. The MTF2 is a polycomb-like (PCL) protein which, in association with the polycomb repressive complex 2 (PRC2), mediates transcriptional repression and regulates several biological processes including cell differentiation (125). The ST18 genes has shown to inhibit colony formation of cancer cells in soft agar and to regulate pro-apoptotic and pro-inflammatory gene expression in fibroblast and in pancreatic B-cells (126, 127). Further studies need to be performed to fully characterize the role of such genes in cervical carcinogenesis.

Few studies investigated the expression of specific circulating miRNAs in distinct stages of cervical carcinoma. Yu et al. detected lower levels of miR-218 in cervical cancer patients sera compared to healthy women matched by age. Importantly such reduction was significantly associated with lymph node metastasis suggesting its potential use as circulating prognostic marker for cervical cancer invasion and metastasis (104). Several other circulating miRNAs have been proposed to be predictive of lymph node metastasis in patients with early stages cervical cancer (39, 97, 128). Among these, promising candidate biomarkers for their limited variations between tumor tissue and serum are represented by miR-1246, miR-20a, miR-2392, miR-3147, miR-3162-5p and miR-4484 (39).

## Deregulated lncRNAs in Cervical Cancer

lncRNAs have the ability to bind proteins, mRNAs or miRNAs, are involved in multiple biological functions and have an important role in cancer development. Several lncRNAs, including HOTAIR, H19, MALT1, CCAT2, SPRY4-IT1, GAS5, CCHE1, MEG3, LET, EBIC, and PVT1, are recognized to play crucial functions in cervical cancer progression, invasion and metastasis as well as in radio-resistance [Table 2; (29, 165)].

**Table 2:** lncRNAs that are reported to have oncogenic or tumor suppressor functions in cervical cancer.

**lncRNAs**	**Function**	**Sponged miRNAs**	**Deregulated pathways in cervical cancer**	**References**
HOTAIR	Oncogenic	miR-22, miR-23b, miR-143-3p	BCL2, PRC2, LSD1, VEGF, mmP-9, mTOR, Notch, Wnt, STAT3, wnt/β-catenin, PI3K/AKT, HPV E7 oncoprotein	(129–139)
H19	Oncogenic	miR-138-5p	IGF2, HPV E6 oncoprotein	(140)
MALAT1	Oncogenic	miR-124, miR-145, miR-206	RBG2, E-cadherin, β-catenin, vimentin, ZO-1, caspase-3, caspase-8, Bax, Bcl-2, and BclxL	(141)
CCAT2	Oncogenic	miR-17-5p, miR-20a	MYC, wnt in colon cancer	(142)
SPRY4-IT1	Oncogenic	miR-101-3p	ZEB1, EMT, E-cadherin, vimentin	(143)
GAS5	Oncosuppressive	miR-106b	IER3	(144)
CCHE1	Oncogenic		PCNA, ERK/MAPK	(145, 146)
MEG3	Oncosuppressive	miR-21-5p		(147)
LET	Oncosuppressive		LIN28	
EBIC	Oncogenic		EZH2, Wnt/β-catenin, E-cadherin	(148, 149)
PVT1	Oncogenic	miR-200, miR-424, miR-195	EZH2, Myc, Nop2, p15, p16, H3K27me3, NF-kB	(150–157)
LINC00675	Oncogenic		Wnt/β-catenin, Bax and GSK-3β Bcl-2	(158)
C5orf66-AS1	Oncogenic	miR-637	RING1	(159)
FAM83H-AS1	Oncogenic		HPV E6, E6-p300	(160)
CCAT1	Oncogenic	miR-181a-5p	MMP14	(161)
NOC2L-4.1	Oncogenic	miR-630	YAP1	(162)
PAX8 AS1	Oncosuppressive		PAX8, NOTCH1 (pancreatic carcinoma)	(163)
RSU1P2	Oncogenic	let-7a	IGF1R, N-myc	(164)

The 2.2 kb HOX transcript antisense intergenic RNA, namely HOTAIR, is a lncRNA encoded by the antisense strand of the HOXC gene located in the chromosome 12 q13.13 (166). Similarly to other lncRNAs, HOTAIR has shown to recruits chromatin-modifying proteins and to affect cancer epigenome modulation (81, 167). Indeed, the HOTAIR 5′ domain binds the zeste homolog 2 (EZH2) and concurs to the silencing of the target gene nemo-like kinase (NLK) (168). In cervical cancer HOTAIR levels have shown to be strictly controlled by the HPV E7 protein (129). Furthermore, HOTAIR has shown to act as sponge for several miRNAs and to cause deregulation of the respective target genes. Indeed, HOTAIR has shown to alter the miR-143-3p/BCL2 axis favoring cervical cancer cell growth (130) and miR-23b/MAPK1 axis contributing to cell proliferation and metastasis (139). Moreover, the over expression of HOTAIR has demonstrated to regulate diverse metabolic functions such as the activation of mTOR pathway in cervical carcinoma cell lines Hela, CaSki, and C33A (131), as well as the activation of Notch-Wnt signaling in SiHa cells (132). HOTAIR expression causes the up regulation of the vascular endothelial growth factor (VEGF), matrix metalloproteinase-9 (MMP-9) and EMT-related genes thus promoting tumor aggressiveness in cervical carcinoma (133). HOTAIR levels have observed to be consistently high in cervical cancer tissues and associated with lymph node metastasis and reduced overall survival (133). Moreover, the HOTAIR levels are higher also in the serum of cervical cancer patients and significantly associated with increased tumor size, lymph vascular space invasion, lymph node metastasis, and reduced survival (132). These data indicated that HOTAIR might represent a novel diagnostic circulating biomarker as well as a promising therapeutic target in cervical cancer. The single nucleotide polymorphism rs920778 T located in the HOTAIR enhancer has shown to be associated with elevated expression of HOTAIR and with cancer susceptibility (169). In addition, the frequency of the rs920778 C HOTAIR allele is reported to be significantly higher in HPV-positive cervical cancer cases while its expression level is observed to be low due to the ability of miR-22 to bind rs920778 C sequence and to suppress the HOTAIR expression (170).

The lncRNA H19, encoded by the H19 gene located in the chromosome loci 11p15.5, is expressed only from the maternally-inherited chromosome (171). It was the first lncRNA identified as a riboregulator and shown to be expressed in fetal tissues and adult muscles as well in many kinds of cancer (172, 173). The lncRNA H19 is modulated by the HPV16 E6 oncoprotein and demonstrated to act as a molecular sponge for miR-138-5p in epithelial cells (160, 174). Kim et al. reported that the lncRNA H19 which is abnormally expressed in cervical cancer may be associated with the cervical cancer progression (140).

The metastasis-associated lung adenocarcinoma transcript 1 (MALAT1), first identified in non-small cell lung cancer, is an 8,000 nucleotides lncRNA located in the chromosome 11q13.1. MALAT1 has shown to promote epigenetic changes and to modulate gene expression, nuclear organization as well as regulation of alternative splicing by acting as decoy for splicing factors (175). It is found largely expressed in many cancer types in relation to the accumulation of aberrant splicing products (176). The MALAT1 is over expressed in cervical cancer cell lines and cancer tissues infected with high risk HPVs (177), acts as a sponge for several miRNAs, including miR-124, miR-145, and miR-206, and favors cervical cancer progression (178). Accordingly, down regulation of MALAT1 in cervical cancer cell lines and in cervical cancer tissues reduces invasion and metastasis through the inhibition of epidermal mesenchymal transition and modulation of the MALAT1-miR-124-RBG2 axis (141). The sponging of miR-145 by MALAT1 has suggested to be involved in the mechanisms of radio-resistance in cervical cancer radiotherapy (178). Moreover, MALAT1 expression has recognized to be an independent prognostic factor in addition to tumor size, FIGO stage, and lymph node metastasis (179). The knockdown of MALAT1 in CaSki cell line caused decrease of cell cycle regulators, such as cyclin D1, cyclin E, and CDK6, leading the cells to accumulate in G1 phase (180).

LncRNA colon cancer-associated transcript 2 (CCAT2) is a 1,752 nucleotide sequence located in the chromosome 8q24 and expressed in microsatellite-stable colorectal cancers (181). Next, CCAT2 has shown to be over expressed and defined as biomarker of poor prognosis in gastric, bladder, non-small cell lung, small cell lung, breast, and ovary cancers as well as in esophageal squamous cell carcinoma and glioma (182). CCAT2 has found to be up regulated in HeLa, CaSki, and SiHa cervical cancer cells as well as in cervical cancer tissues (183). The inhibition of CCAT2 expression by siRNA in cervical cancer cells has shown to suppress cell proliferation. In cervical cancer patients the high expression of CCAT2 is correlated to advanced FIGO stage, lymph node metastasis, deep cervical invasion and reduced survival (183). However, the molecular mechanism mediating the activity of CCAT2 in cervical cancer remain uncharacterized.

SPRY4 intronic transcript 1 (SPRY4-IT1) derives from the intron two of the SPRY4 gene and has shown to act as an oncogenic factor or a tumor suppressor in different cancer types (19, 184). Indeed, it is reported to be over expressed in melanoma, non-small cell lung, esophageal cancer and under expressed in gastric cancer (19, 185, 186). The expression levels of SPRY4-IT1 have been found higher in cervical cancer than in normal tissues and associated with advanced clinical stages and shorter overall survival of cervical cancer patients (187). More recently, the silencing of SPRY4-IT1 in cervical cancer cell lines has shown to inhibit migration and invasion through the SPYR4-IT1/miR-101-3p/ZEB1 axis. This effect is associated with suppression of EMT changes, increased E-cadherin levels and decreased the N-cadherin and vimentin expression (143).

Growth arrest-specific transcript 5 (GAS5) is 651 nucleotide lncRNA encoded by a sequence located in the chromosome 1q25 (188). GAS5 has tumor suppressor activity in several cancer types (189). Accordingly, its decreased expression has a strong association with tumor development and worse clinical outcome in cervical cancer patients (190). Moreover, inhibition of GAS5 in cervical cancer cells has shown to increase the proliferation, migration and invasion confirming its oncosuppressor activity in the cervical cancer progression (190). Gao et al. reported that GAS5 acts as miR-106b sponge causing up regulation of IER3 expression and enhanced radio-sensitivity of cervical cancer cells (144).

Cervical carcinoma high-expressed 1 (CCHE1) lncRNA is 2,500 nucleotide sequence transcribed from a region located in the chromosome 10. It is over expressed in cervical cancer in association with advanced tumor stages, increasing tumor size, invasion and poor prognosis (191). The CCHE1 has demonstrated to bind and stabilize the mRNA of proliferating cell nuclear antigen (PCNA) thus promoting its over expression and increased cervical cancer cell proliferation (145). Peng and Fan, demonstrated that CCHE1 inhibition causes inactivation of the ERK/MAPK pathway, growth arrest and apoptosis (146).

Maternally expressed gene (MEG3) is a 1,600 nucleotide lncRNA derived from the DLK1-MEG3 locus located on the chromosomal region 14q32.3, first identified as the ortholog of gene trap locus 2 (Gtl2) in mice (192). MEG3 is expressed in many normal tissues and its loss has been reported in many cancer types. The over expression of MEG3 can inhibit proliferation and increase apoptosis in cancer cells either in a p53 dependent or independent manner (193). Expression of MEG3 is low both in cervical cancer tissues and in cervical cancer cell lines. Increased levels of MEG3 in cervical cancer cell lines have shown to inhibit cell proliferation, induce cell cycle arrest and cause apoptosis (194). MEG3 has shown to act as a cancer suppressor through its ability to down regulate the miR-21-5p levels in cervical cancer cell lines (147). Indeed, knockdown of MEG3 in HeLa and CaSki cells induced significant up regulation of miR-21-5p expression (147). Thus, could act as a tumor suppressor able to inhibit tumor growth in cervical cancer.

LncRNA-Low Expression in Tumor (LET), is a 2,600 nucleotide long RNA transcribed from a region located in the chromosome locus 15q24.1. It is down-regulated in hepatocellular, gallbladder, esophageal and cervical carcinoma (182, 195). In cervical cancer patients has been reported a significant correlation between LET expression levels and clinico-pathological parameters suggesting that it might be considered a prognostic biomarker if confirmed by more clinical studies (196).

EZH2-binding lncRNA (EBIC) is a 1,500 nucleotide RNA encoded by a sequence located in the chromosome locus 12q22. The over expression of EBIC has shown to deregulate the Wnt/β-catenin signaling pathway and to promote cell proliferation, invasion and migration as well as to increase cisplatin resistance in ovarian cancer (148). EBIC has been described as an oncogenic lncRNA also in cervical cancer, indeed it was demonstrated to promote tumor cell invasion by binding to EZH2 and inhibiting E-cadherin expression (149).

Plasmacytoma variant translocation 1 (PVT1) is a highly conserved lncRNA, which is located downstream of MYC gene and is frequently co-amplified with MYC in several cancer types (150–152, 193). Iden et al. utilized siRNA and LNA-mediated knockdown to analyze the effect of reduced levels of PVT1 in cervical cancer cells obtaining inhibition of cell proliferation, migration, invasion and cisplatin resistance (153). Furthermore, PVT1 has shown to decrease miR-195 and miR-200b expression in cervical cancer either by enhancing histone H3K27me3 in the miR-195 and miR-200b promoters or by direct binding of miR-195 and miR-200b (154, 155). MiR-195 is associated with epithelial-mesenchymal transition and chemo resistance, whereas miR-200b is associated with cell proliferation, invasion and migration of cervical cancer cells. Yang et al. reported that serum levels of PVT1 positively correlate with PVT1 expression in cancer tissues indicating that it could serve as a biomarker for diagnosis of cervical cancer (156).

Along with the above-mentioned lncRNAs, many other novel sequences, such as LINC00675, C5orf66-AS1, FAM83H-AS1, CCAT1, NOC2L-4.1, PAX8 AS1, and RSU1P2, have identified as playing multiple roles in cervical tumorigenesis (Table 2). Their ability to sponge specific miRNAs and to deregulate metabolic pathways have been mainly investigated in cell culture models. Therefore, clinical studies are needed to determine the possible use of these novel lncRNAs as biomarkers for diagnosis and prognosis of cervical cancer.

## Circular RNAs in Cervical Cancer

CircRNAs are highly expressed in many cancer types, such as breast, lung and colorectal cancer (197–200). Several circRNAs are abnormally expressed in cervical cancer tissues as well as derived cell lines and contribute to tumorigenesis mostly by sequestering miRNAs [Table 3; (34)]. Among these, hsa_circ_0141539 (circRNA-000284), encoded by the Clorf116 gene, was found significantly over expressed in cervical cancer tissues compared to adjacent non-tumor tissues and directly associated with tumor size, FIGO stage as well as myometrium invasion (202). The hsa_circ_0141539 is able to sponge miR-518d-5p/519-5p causing increased expression of the target gene CBX8 and promotion of malignant transformation of cervical cells (202). This circRNA was also demonstrated to sponge miR-506 causing an increased expression of Snail-2 which is a direct target of miR-506 (201). Silencing of hsa_circ_0141539 has been proposed as a novel treatment strategy for cervical cancer patients.

**Table 3:** CircRNA that were reported to be deregulated in cervical neoplasia.

**CircRNAs**	**Targets**	**Effect in cervical cancer and/or cell lines**	**References**
Hsa_circ_000284	miR-506, Snail-2	Promote proliferation and invasion	(201)
Hsa_circ_0141539	miR-518d-5p, miR-519-5p, CBX8	Promote proliferation, migration and invasion	(202)
Hsa_circ_0023404	miR-136, TFCP2, YAP	Promote proliferation and invasion	(203)
Hsa_circ_0018289	miR-497	Promote cell proliferation, migration and invasion	(35)
Hsa_circ_0000263	miR-150-5p, MDM4, p53	Promote cell proliferation, migration	(204)
Hsa_circ_0001445	miR-620	Suppress proliferation and invasion	(205)
Hsa_circRNA_101996	miR-8075, TPX2		
Hsa_circ_0031288	HuR, PABPN1	Decrease cell proliferation	(206)
Hsa_circ_0004015	miR-1183, and PDPK1	Promote cell migration, angiogenesis and radio-resistance	(207)
CircATP8A2	miR-433, EGFR	Promote cervical cancer progression	(208)
Circ_0067934	miR-545	Promote cervical cancer progression	(209)
CircEIF4G2	miR-218, HOXA1	Promote cell proliferation and migration	(210)
CircCLK3	miR-320a, FoxM1	Promote cervical cancer progression	(211)
CircE7	pRb	HPV encoded circular E7 RNA promoting cell transformation	(212)

The circRNA Hsa_circ_0023404, derived from the RNF121 gene located on the chromosome 11, was significantly over expressed in cervical cancer and associated with poor prognosis (203). Hsa_circ_0023404 has shown to sequester miR-136 thus promoting over expression of target gene TFCP2, consequent activation of YAP signaling pathway and consequent cervical cancer development and progression (203). More recently, Guo et al. reported a novel biological effect of hsa_circ_0023404 relying on the subtraction of miR-5047 with consequent induction of VEGFA expression and increased cervical cancer metastasis and chemoresistance. Moreover, hsa_circ_0023404 and VEGFA were found concordantly up regulated in cervical tumors, while miR-5047 was under expressed (213).

Gao et al. performed a microarray analysis of 35 cervical cancer cases and identified 45 significantly up regulated circRNAs with hsa_circ_0018289 as the most deregulated (35). Hsa_circ_0018289 was observed to directly bind miR-497 causing increased cell proliferation, migration and invasion of cervical cancer cells (35). Thus, hsa_circ_0018289, similarly to other circRNA, has demonstrated to have an oncogenic role in cervical cancer development. Cai et al. reported that also hsa_circ_0000263 has an oncogenic role being significantly up regulated in cervical cancer cells, able to bind miR-150-5p and to promote cell proliferation and migration through the hsa_circ_0000263/miR-150-5p/MDM4/p53 regulatory network affecting p53 activity (204). Conversely, the hsa_circ_0001445 (circSMARCA5) is able to sponge the miR-620 and to act as an oncosuppressor being its expression decreased in cervical cancer cells and its over expression causing inhibition of cell proliferation, invasion and migration (205).

Very recently, Song et al. by performing *in silico* analyses detected higher expression of hsa_circRNA_101996 in cervical cancer and observed a correlation with tumor stage, tumor size, lymph node metastasis, and poor outcomes (214). The oncogenic activity of hsa_circRNA_101996 has shown to be mediated by sequestration of miR-8075 and deregulation of TPX2 gene expression which represents a new mechanism of cervical cancer development (214). Others up regulated circRNAs in cervical cancer cells are circATP8A2 promoting cervical cancer progression through the circATP8A2/miR-433/EGFR axis, and circ_0067934 which binds miR-545 and is associated with advanced cancer stage, lymph node metastasis, and poor prognosis (208, 209). Moreover, Mao et al. identified the circEIF4G2 as over expressed in cervical cancer cells and able to interact with miR-218 causing enhancement of cervical cancer progression through the circEIF4G2-miR-218/HOXA1 axis (210). Recently, the circCLK3, which is a novel circRNA found over expressed in cervical carcinoma, has demonstrated to sponge miR-320a and to abolish its ability to suppress FoxM1 transcription factor expression as well as to promote cell proliferation, epidermal mesenchymal transition, migration, and invasion of cervical cancer cells (211). A recent study evaluating the expression profile of circRNAs by high-throughput RNA sequencing in three HPV16 positive cervical cancer cases identified 99 deregulated circRNAs (58 over expressed and 41 under expressed circRNAs) of which 44 have not been previously described and their role in cancer has not yet established (215). All these findings clearly show that many circRNAs play key roles in cervical cancer development and that they can be used as novel diagnostic and prognostic biomarkers as well as for targeted therapies. However, more studies are needed to establish their significance as biomarkers of early disease and clinical outcome indicators.

High risk HPVs have recently demonstrated to encode a circRNA encompassing the E7 oncogene (circE7) (212). Such viral circRNA has shown to be modified by the N6-methyladenosine (m6A), to localize in the cytoplasm, to associate with polysomes, and to produce E7 oncoprotein. CircE7 is expressed in HPV16 positive CaSki cells and its targeted disruption causes reduction of E7 oncoprotein levels and cell growth inhibition (212). These results provide evidence of a new molecular mechanism of viral related tumorigenesis in HPV-related human malignancies and the possibility to search for new types of viral nucleic acids in order to discriminate progressing vs. regressing cervical lesions.

## Conclusion

The discovery of aberrantly expressed miRNAs, lncRNAs, and circRNAs in cervical cancer have defined new molecular mechanisms of cervical cancer tumorigenesis and provided considerable opportunities for translating the non-coding RNAs research into clinical settings. Data from emerging studies clearly highlight the significance of specific miRNA signatures in the diagnosis and prognosis of cervical cancer. Particularly, miRNAs with oncogenic potential differently expressed in different stages of cervical cancer and those associated with high risk HPV infection might represent promising biomarkers for oncologic screening and cancer recurrence monitoring. Indeed, the analysis of specific miRNA panels have demonstrated a greater diagnostic value, higher sensitivity and specificity, in patients with cervical lesions and cancer. More importantly, circulating miRNAs appear valuable for the early detection of cervical cancer and for monitoring the clinical outcome of advanced cancer considering that the majority of cancer associated deaths are caused by metastases to distant organs. In addition, miRNA testing may reduce the need for invasive cervical biopsies and are useful in predicting the prognosis of cervical cancer. However, larger studies are required to validate miRNA assays as diagnostic marker in comparison with cytology and HPV screening methods. Several molecules able to target oncogenic miRNAs have been developed such as the synthetic antisense oligonucleotides encoding miRNA complementary sequences. Hypomethylation agents such as decitabine or 5-azacitidine have also shown effective to induce epigenetic silencing of miRNAs and they are the approved treatments for downregulating the expression of several non-coding RNAs and mRNAs in myelodysplastic syndromes. Therefore, such therapeutic approaches, although in the early stage of clinical translation, might become effective drugs providing insight into the treatment of all types of cancers.

lncRNAs are important as potential biomarkers for cervical prognosis, invasion, metastasis, chemo-radio-resistance. Several lncRNAs can be detected in the serum and other biological fluids. Indeed, circulating HOTAIR, MALAT1, and MEG3 transcripts were found significantly higher in cancer patients compared to healthy subjects suggesting their potential as diagnostic biomarkers. Moreover, the increased HOTAIR levels in the serum of cervical cancer patients is also indicative of the metastatic tumor phase, adenocarcinoma, lymphatic node metastasis and tumor recurrence. The molecular mechanisms of lncRNAs in cervical cancer need further characterization. Particularly the interplay with miRNAs and circRNAs needs to be fully discovered. Inhibition of the oncogenic activity of lncRNAs is also in the early phase of research since no RNA-interference drugs have been approved for clinical use.

CircRNAs have been recently discovered and their role in cervical cancer pathogenesis recognized. Massive parallel sequencing and bioinformatics techniques allowed uncovering many circRNAs differentially expressed in cervical cancer tissues compared to normal cells suggesting that they have relevant roles in this cancer type. Their activity is mainly mediated by the sponging of specific miRNAs. The study of circRNAs potentially represents a new promising strategy for diagnosis and treatment of cervical cancer. However, the characterization of biological functions of circRNAs in cancer cells is in a nascent stage and most activities have not been fully elucidated.

More studies mainly based on high-throughput sequencing technologies, which simultaneously evaluate the network of miRNAs, lncRNAs, and circRNAs are needed to further reveal the complexity of the interplay between diverse classes of non-coding RNAs and deregulation of new actionable metabolic pathways for treatment of cervical cancer.

## Author Contributions

MT, RF, LB, CA, NS, AC, FP, AT, and FB contributed to the writing and editing of the manuscript.

### Conflict of Interest

The authors declare that the research was conducted in the absence of any commercial or financial relationships that could be construed as a potential conflict of interest.

## References

[1] Bray F Ferlay J Soerjomataram I Siegel RL Torre LA Jemal A. Global cancer statistics 2018: GLOBOCAN estimates of incidence and mortality worldwide for 36 cancers in 185 countries. CA Cancer J Clin. (2018) 68:394–424. 10.3322/caac.21492 30207593

[2] Schiffman M Wentzensen N Wacholder S Kinney W Gage JC Castle PE. Human papillomavirus testing in the prevention of cervical cancer. J Natl Cancer Inst. (2011) 103:368–83. 10.1093/jnci/djq562 21282563 PMC3046952

[3] Ronco G Dillner J Elfstrom KM Tunesi S Snijders PJ Arbyn M. Efficacy of HPV-based screening for prevention of invasive cervical cancer: follow-up of four European randomised controlled trials. Lancet. (2014) 383:524–32. 10.1016/S0140-6736(13)62218-7 24192252

[4] Tornesello ML Annunziata C Tornesello AL Buonaguro L Buonaguro FM. Human oncoviruses and p53 tumor suppressor pathway deregulation at the origin of human cancers. Cancers. (2018) 10:E213. 10.3390/cancers10070213 29932446 PMC6071257

[5] Yeo-Teh NSL Ito Y Jha S. High-risk human papillomaviral oncogenes E6 and E7 target key cellular pathways to achieve oncogenesis. Int J Mol Sci. (2018) 19:E1706. 10.3390/ijms19061706 29890655 PMC6032416

[6] Venuti A Paolini F Nasir L Corteggio A Roperto S Campo MS. Papillomavirus E5: the smallest oncoprotein with many functions. Mol Cancer. (2011) 10:140. 10.1186/1476-4598-10-140 22078316 PMC3248866

[7] Scott ML Coleman DT Kelly KC Carroll JL Woodby B Songock WK. Human papillomavirus type 16 E5-mediated upregulation of Met in human keratinocytes. Virology. (2018) 519:1–11. 10.1016/j.virol.2018.03.021 29609071 PMC5971161

[8] Wright AA Howitt BE Myers AP Dahlberg SE Palescandolo E Van HP. Oncogenic mutations in cervical cancer: genomic differences between adenocarcinomas and squamous cell carcinomas of the cervix. Cancer. (2013) 119:3776–83. 10.1002/cncr.28288 24037752 PMC3972000

[9] Tornesello ML Annunziata C Buonaguro L Losito S Greggi S Buonaguro FM. TP53 and PIK3CA gene mutations in adenocarcinoma, squamous cell carcinoma and high-grade intraepithelial neoplasia of the cervix. J Transl Med. (2014) 12:255. 10.1186/s12967-014-0255-5 25220666 PMC4174264

[10] Tornesello ML Buonaguro L Buonaguro FM. Mutations of the TP53 gene in adenocarcinoma and squamous cell carcinoma of the cervix: a systematic review. Gynecol Oncol. (2013) 128:442–8. 10.1016/j.ygyno.2012.11.017 23168175

[11] Wingo SN Gallardo TD Akbay EA Liang MC Contreras CM Boren T. Somatic LKB1 mutations promote cervical cancer progression. PLoS ONE. (2009) 4:e5137. 10.1371/journal.pone.0005137 19340305 PMC2660434

[12] Ojesina AI Lichtenstein L Freeman SS Pedamallu CS Imaz-Rosshandler I Pugh TJ. Landscape of genomic alterations in cervical carcinomas. Nature. (2014) 506:371–5. 10.1038/nature12881 24390348 PMC4161954

[13] Cancer Genome Atlas Research Network Albert Einstein College of Medicine Analytical Biological Services Barretos Cancer Hospital Baylor College of Medicine Beckman Research Institute of City of Hope Integrated genomic and molecular characterization of cervical cancer. Nature. (2017) 543:378–84. 10.1038/nature21386 28112728 PMC5354998

[14] Annunziata C Pezzuto F Greggi S Ionna F Losito S Botti G. Distinct profiles of TERT promoter mutations and telomerase expression in head and neck cancer and cervical carcinoma. Int J Cancer. (2018) 143:1153–61. 10.1002/ijc.31412 29603728

[15] Peng L Yuan X Jiang B Tang Z Li GC. lncRNAs: key players and novel insights into cervical cancer. Tumour Biol. (2016) 37:2779–88. 10.1007/s13277-015-4663-9 26715267

[16] Reshmi G Pillai MR. Beyond HPV: oncomirs as new players in cervical cancer. FEBS Lett. (2008) 582:4113–6. 10.1016/j.febslet.2008.11.011 19032954

[17] Yi Y Liu Y Wu W Wu K Zhang W. Reconstruction and analysis of circRNAmiRNAmRNA network in the pathology of cervical cancer. Oncol Rep. (2019) 41:2209–25. 10.3892/or.2019.7028 30816541 PMC6412533

[18] Lai EC. Micro RNAs are complementary to 3' UTR sequence motifs that mediate negative post-transcriptional regulation. Nat Genet. (2002) 30:363–4. 10.1038/ng865 11896390

[19] Xie M Nie FQ Sun M Xia R Liu YW Zhou P. Decreased long noncoding RNA SPRY4-IT1 contributing to gastric cancer cell metastasis partly via affecting epithelial-mesenchymal transition. J Transl Med. (2015) 13:250. 10.1186/s12967-015-0595-9 26238992 PMC4522960

[20] Liu H Lei C He Q Pan Z Xiao D Tao Y. Nuclear functions of mammalian MicroRNAs in gene regulation, immunity and cancer. Mol Cancer. (2018) 17:64. 10.1186/s12943-018-0765-5 29471827 PMC5822656

[21] Friedlander MR Lizano E Houben AJ Bezdan D Banez-Coronel M Kudla G. Evidence for the biogenesis of more than 1,000 novel human microRNAs. Genome Biol. (2014) 15:R57–15. 10.1186/gb-2014-15-4-r57 24708865 PMC4054668

[22] Jens M Rajewsky N. Competition between target sites of regulators shapes post-transcriptional gene regulation. Nat Rev Genet. (2015) 16:113–26. 10.1038/nrg3853 25488579

[23] Romano G Veneziano D Acunzo M Croce CM. Small non-coding RNA and cancer. Carcinogenesis. (2017) 38:485–91. 10.1093/carcin/bgx026 28449079 PMC6248440

[24] He Y Lin J Ding Y Liu G Luo Y Huang M. A systematic study on dysregulated microRNAs in cervical cancer development. Int J Cancer. (2016) 138:1312–27. 10.1002/ijc.29618 26032913

[25] Hong S Cheng S Songock W Bodily J Laimins LA. Suppression of MicroRNA 424 levels by human papillomaviruses is necessary for differentiation-dependent genome amplification. J Virol. (2017) 91:e01712–17. 10.1128/JVI.01712-17 28978708 PMC5709607

[26] Ulitsky I Bartel DP. lincRNAs: genomics, evolution, and mechanisms. Cell. (2013) 154:26–46. 10.1016/j.cell.2013.06.020 23827673 PMC3924787

[27] Sun Q Hao Q Prasanth KV. Nuclear long noncoding RNAs: key regulators of gene expression. Trends Genet. (2018) 34:142–57. 10.1016/j.tig.2017.11.005 29249332 PMC6002860

[28] Marchese FP Raimondi I Huarte M. The multidimensional mechanisms of long noncoding RNA function. Genome Biol. (2017) 18:206. 10.1186/s13059-017-1348-2 29084573 PMC5663108

[29] Dong J Su M Chang W Zhang K Wu S Xu T. Long non-coding RNAs on the stage of cervical cancer (review). Oncol Rep. (2017) 38:1923–31. 10.3892/or.2017.5905 28849103

[30] Sharma S Munger K. Expression of the cervical carcinoma expressed PCNA regulatory (CCEPR) long noncoding RNA is driven by the human papillomavirus E6 protein and modulates cell proliferation independent of PCNA. Virology. (2018) 518:8–13. 10.1016/j.virol.2018.01.031 29427865 PMC5911229

[31] He H Liu X Liu Y Zhang M Lai Y Hao Y. Human papillomavirus E6/E7 and long noncoding RNA TMPOP2 mutually upregulated gene expression in cervical cancer cells. J Virol. (2019) 93:e01808–18. 10.1128/JVI.01808-18 30728257 PMC6450114

[32] Haddad G Lorenzen JM. Biogenesis and function of circular RNAs in health and in disease. Front Pharmacol. (2019) 10:428. 10.3389/fphar.2019.00428 31080413 PMC6497739

[33] Bach DH Lee SK Sood AK. Circular RNAs in cancer. Mol Ther Nucleic Acids. (2019) 16:118–29. 10.1016/j.omtn.2019.02.005 30861414 PMC6411617

[34] Chaichian S Shafabakhsh R Mirhashemi SM Moazzami B Asemi Z. Circular RNAs: a novel biomarker for cervical cancer. J Cell Physiol. (2019) 235:718–24. 10.1002/jcp.29009 31240697

[35] Gao YL Zhang MY Xu B Han LJ Lan SF Chen J. Circular RNA expression profiles reveal that hsa_circ_0018289 is up-regulated in cervical cancer and promotes the tumorigenesis. Oncotarget. (2017) 8:86625–33. 10.18632/oncotarget.21257 29156822 PMC5689712

[36] Wilting SM Snijders PJ Verlaat W Jaspers A van de Wiel MA van Wieringen WN. Altered microRNA expression associated with chromosomal changes contributes to cervical carcinogenesis. Oncogene. (2013) 32:106–16. 10.1038/onc.2012.20 22330141

[37] Pardini B De MD Francavilla A Di GC Ronco G Naccarati A. MicroRNAs as markers of progression in cervical cancer: a systematic review. BMC Cancer. (2018) 18:696. 10.1186/s12885-018-4590-4 29945565 PMC6020348

[38] Gocze K Gombos K Juhasz K Kovacs K Kajtar B Benczik M. Unique microRNA expression profiles in cervical cancer. Anticancer Res. (2013) 33:2561–7. 23749909

[39] Chen J Yao D Li Y Chen H He C Ding N. Serum microRNA expression levels can predict lymph node metastasis in patients with early-stage cervical squamous cell carcinoma. Int J Mol Med. (2013) 32:557–67. 10.3892/ijmm.2013.1424 23799609 PMC3782554

[40] Zhang J Zheng F Yu G Yin Y Lu Q. miR-196a targets netrin 4 and regulates cell proliferation and migration of cervical cancer cells. Biochem Biophys Res Commun. (2013) 440:582–8. 10.1016/j.bbrc.2013.09.142 24120501

[41] Hou T Ou J Zhao X Huang X Huang Y Zhang Y. MicroRNA-196a promotes cervical cancer proliferation through the regulation of FOXO1 and p27Kip1. Br J Cancer. (2014) 110:1260–8. 10.1038/bjc.2013.829 24423924 PMC3950858

[42] Xu XM Wang XB Chen MM Liu T Li YX Jia WH. MicroRNA-19a and−19b regulate cervical carcinoma cell proliferation and invasion by targeting CUL5. Cancer Lett. (2012) 322:148–58. 10.1016/j.canlet.2012.02.038 22561557

[43] Liu C Lin J Li L Zhang Y Chen W Cao Z. HPV16 early gene E5 specifically reduces miRNA-196a in cervical cancer cells. Sci Rep. (2015) 5:7653. 10.1038/srep07653 25563170 PMC4288222

[44] Wang N Zhou Y Zheng L Li H. MiR-31 is an independent prognostic factor and functions as an oncomir in cervical cancer via targeting ARID1A. Gynecol Oncol. (2014) 134:129–37. 10.1016/j.ygyno.2014.04.047 24793973

[45] Yao T Lin Z. MiR-21 is involved in cervical squamous cell tumorigenesis and regulates CCL20. Biochim Biophys Acta. (2012) 1822:248–60. 10.1016/j.bbadis.2011.09.018 22001440

[46] Zhang S Liu F Mao X Huang J Yang J Yin X. Elevation of miR-27b by HPV16 E7 inhibits PPARgamma expression and promotes proliferation and invasion in cervical carcinoma cells. Int J Oncol. (2015) 47:1759–66. 10.3892/ijo.2015.3162 26397063

[47] Zhou C Shen L Mao L Wang B Li Y Yu H. miR-92a is upregulated in cervical cancer and promotes cell proliferation and invasion by targeting FBXW7. Biochem Biophys Res Commun. (2015) 458:63–9. 10.1016/j.bbrc.2015.01.066 25623537

[48] Lao G Liu P Wu Q Zhang W Liu Y Yang L. Mir-155 promotes cervical cancer cell proliferation through suppression of its target gene LKB1. Tumour Biol. (2014) 35:11933–8. 10.1007/s13277-014-2479-7 25155037

[49] Yang L Wang YL Liu S Zhang PP Chen Z Liu M. miR-181b promotes cell proliferation and reduces apoptosis by repressing the expression of adenylyl cyclase 9 (AC9) in cervical cancer cells. FEBS Lett. (2014) 588:124–30. 10.1016/j.febslet.2013.11.019 24269684

[50] Zheng W Liu Z Zhang W Hu X. miR-31 functions as an oncogene in cervical cancer. Arch Gynecol Obstet. (2015) 292:1083–9. 10.1007/s00404-015-3713-2 25894339

[51] Kang HW Wang F Wei Q Zhao YF Liu M Li X. miR-20a promotes migration and invasion by regulating TNKS2 in human cervical cancer cells. FEBS Lett. (2012) 586:897–904. 10.1016/j.febslet.2012.02.020 22449978

[52] Xie H Zhao Y Caramuta S Larsson C Lui WO. miR-205 expression promotes cell proliferation and migration of human cervical cancer cells. PLoS ONE. (2012) 7:e46990. 10.1371/journal.pone.0046990 23056551 PMC3463520

[53] Zhao S Yao D Chen J Ding N Ren F. MiR-20a promotes cervical cancer proliferation and metastasis *in vitro* and *in vivo*. PLoS ONE. (2015) 10:e0120905. 10.1371/journal.pone.0120905 25803820 PMC4372287

[54] Wu X Xi X Yan Q Zhang Z Cai B Lu W. MicroRNA-361–5p facilitates cervical cancer progression through mediation of epithelial-to-mesenchymal transition. Med Oncol. (2013) 30:751. 10.1007/s12032-013-0751-0 24158756

[55] Wang LQ Zhang Y Yan H Liu KJ Zhang S. MicroRNA-373 functions as an oncogene and targets YOD1 gene in cervical cancer. Biochem Biophys Res Commun. (2015) 459:515–20. 10.1016/j.bbrc.2015.02.138 25747718

[56] Sun Y Zhang B Cheng J Wu Y Xing F Wang Y. MicroRNA-222 promotes the proliferation and migration of cervical cancer cells. Clin Invest Med. (2014) 37:E131. 10.25011/cim.v37i3.21380 24895988

[57] Xie H Lee L Scicluna P Kavak E Larsson C Sandberg R. Novel functions and targets of miR-944 in human cervical cancer cells. Int J Cancer. (2015) 136:E230–E241. 10.1002/ijc.29160 25156441 PMC4277326

[58] Lee JW Choi CH Choi JJ Park YA Kim SJ Hwang SY. Altered MicroRNA expression in cervical carcinomas. Clin Cancer Res. (2008) 14:2535–42. 10.1158/1078-0432.CCR-07-1231 18451214

[59] Nagamitsu Y Nishi H Sasaki T Takaesu Y Terauchi F Isaka K. Profiling analysis of circulating microRNA expression in cervical cancer. Mol Clin Oncol. (2016) 5:189–94. 10.3892/mco.2016.875 27330796 PMC4906571

[60] Sharma S Hussain S Soni K Singhal P Tripathi R Ramachandran VG. Novel MicroRNA signatures in HPV-mediated cervical carcinogenesis in Indian women. Tumour Biol. (2016) 37:4585–95. 10.1007/s13277-015-4248-7 26508022

[61] Jia W Wu Y Zhang Q Gao GE Zhang C Xiang Y. Expression profile of circulating microRNAs as a promising fingerprint for cervical cancer diagnosis and monitoring. Mol Clin Oncol. (2015) 3:851–8. 10.3892/mco.2015.560 26171195 PMC4486870

[62] Cheung TH Man KN Yu MY Yim SF Siu NS Lo KW. Dysregulated microRNAs in the pathogenesis and progression of cervical neoplasm. Cell Cycle. (2012) 11:2876–84. 10.4161/cc.21278 22801550

[63] Yamamoto N Kinoshita T Nohata N Itesako T Yoshino H Enokida H. Tumor suppressive microRNA-218 inhibits cancer cell migration and invasion by targeting focal adhesion pathways in cervical squamous cell carcinoma. Int J Oncol. (2013) 42:1523–32. 10.3892/ijo.2013.1851 23483249 PMC3661225

[64] Liu L Yu X Guo X Tian Z Su M Long Y. miR-143 is downregulated in cervical cancer and promotes apoptosis and inhibits tumor formation by targeting Bcl-2. Mol Med Rep. (2012) 5:753–60. 10.3892/mmr.2011.696 22160209

[65] Liu S Song L Yao H Zhang L Xu D Gao F Li Q. MiR-375 is epigenetically downregulated by HPV-16 E6 mediated DNMT1 upregulation and modulates EMT of cervical cancer cells by suppressing lncRNA MALAT1. PLoS ONE. (2016) 11:e0163460. 10.1371/journal.pone.0163460 27658300 PMC5033370

[66] Wen Z Lei Z Jin-An M Xue-Zhen L Xing-Nan Z Xiu-Wen D. The inhibitory role of miR-214 in cervical cancer cells through directly targeting mitochondrial transcription factor A (TFAM). Eur J Gynaecol Oncol. (2014) 35:676–82. 10.12892/ejgo24572014 25556274

[67] Qiang R Wang F Shi LY Liu M Chen S Wan HY. Plexin-B1 is a target of miR-214 in cervical cancer and promotes the growth and invasion of HeLa cells. Int J Biochem Cell Biol. (2011) 43:632–41. 10.1016/j.biocel.2011.01.002 21216304

[68] Peng RQ Wan HY Li HF Liu M Li X Tang H. MicroRNA-214 suppresses growth and invasiveness of cervical cancer cells by targeting UDP-N-acetyl-alpha-D-galactosamine:polypeptide N-acetylgalactosaminyltransferase 7. J Biol Chem. (2012) 287:14301–9. 10.1074/jbc.M111.337642 22399294 PMC3340176

[69] Yang Z Chen S Luan X Li Y Liu M Li X. MicroRNA-214 is aberrantly expressed in cervical cancers and inhibits the growth of HeLa cells. IUBMB Life. (2009) 61:1075–82. 10.1002/iub.252 19859982

[70] Zhu X Er K Mao C Yan Q Xu H Zhang Y. miR-203 suppresses tumor growth and angiogenesis by targeting VEGFA in cervical cancer. Cell Physiol Biochem. (2013) 32:64–73. 10.1159/000350125 23867971

[71] Mao L Zhang Y Mo W Yu Y Lu H. BANF1 is downregulated by IRF1-regulated microRNA-203 in cervical cancer. PLoS ONE. (2015) 10:e0117035. 10.1371/journal.pone.0117035 25658920 PMC4319761

[72] Sun J Ji J Huo G Song Q Zhang X. miR-182 induces cervical cancer cell apoptosis through inhibiting the expression of DNMT3a. Int J Clin Exp Pathol. (2015) 8:4755–63. 10.1007/978-1-4939-1692-4 26191165 PMC4503037

[73] Song X Shi B Huang K Zhang W. miR-133a inhibits cervical cancer growth by targeting EGFR. Oncol Rep. (2015) 34:1573–80. 10.3892/or.2015.4101 26134491

[74] Zhao Q Zhai YX Liu HQ Shi YA Li XB. MicroRNA-491–5p suppresses cervical cancer cell growth by targeting hTERT. Oncol Rep. (2015) 34:979–86. 10.3892/or.2015.4013 26034994

[75] Deng B Zhang Y Zhang S Wen F Miao Y Guo K. MicroRNA-142–3p inhibits cell proliferation and invasion of cervical cancer cells by targeting FZD7. Tumour Biol. (2015) 36:8065–73. 10.1007/s13277-015-3483-2 25976503

[76] Zhou WY Chen JC Jiao TT Hui N Qi X. MicroRNA-181 targets Yin Yang 1 expression and inhibits cervical cancer progression. Mol Med Rep. (2015) 11:4541–6. 10.3892/mmr.2015.3324 25672374

[77] Zhou C Li G Zhou J Han N Liu Z Yin J. miR-107 activates ATR/Chk1 pathway and suppress cervical cancer invasion by targeting MCL1. PLoS ONE. (2014) 9:e111860. 10.1371/journal.pone.0111860 25386925 PMC4227659

[78] Wan HY Li QQ Zhang Y Tian W Li YN Liu M. MiR-124 represses vasculogenic mimicry and cell motility by targeting amotL1 in cervical cancer cells. Cancer Lett. (2014) 355:148–58. 10.1016/j.canlet.2014.09.005 25218344

[79] Liang X Liu Y Zeng L Yu C Hu Z Zhou Q. miR-101 inhibits the G1-to-S phase transition of cervical cancer cells by targeting Fos. Int J Gynecol Cancer. (2014) 24:1165–72. 10.1097/IGC.0000000000000187 24987920

[80] Dong J Sui L Wang Q Chen M Sun H. MicroRNA-26a inhibits cell proliferation and invasion of cervical cancer cells by targeting protein tyrosine phosphatase type IVA 1. Mol Med Rep. (2014) 10:1426–32. 10.3892/mmr.2014.2335 24939702

[81] Luo M Li Z Wang W Zeng Y Liu Z Qiu J. Long non-coding RNA H19 increases bladder cancer metastasis by associating with EZH2 and inhibiting E-cadherin expression. Cancer Lett. (2013) 333:213–21. 10.1016/j.canlet.2013.01.033 23354591

[82] Xin JX Yue Z Zhang S Jiang ZH Wang PY Li YJ. miR-99 inhibits cervical carcinoma cell proliferation by targeting TRIB2. Oncol Lett. (2013) 6:1025–30. 10.3892/ol.2013.1473 24137458 PMC3796436

[83] Wang L Chang L Li Z Gao Q Cai D Tian Y. miR-99a and−99b inhibit cervical cancer cell proliferation and invasion by targeting mTOR signaling pathway. Med Oncol. (2014) 31:934. 10.1007/s12032-014-0934-3 24668416

[84] Wen SY Lin Y Yu YQ Cao SJ Zhang R Yang XM. miR-506 acts as a tumor suppressor by directly targeting the hedgehog pathway transcription factor Gli3 in human cervical cancer. Oncogene. (2015) 34:717–25. 10.1038/onc.2014.9 24608427

[85] Cui F Li X Zhu X Huang L Huang Y Mao C. MiR-125b inhibits tumor growth and promotes apoptosis of cervical cancer cells by targeting phosphoinositide 3-kinase catalytic subunit delta. Cell Physiol Biochem (2012) 30:1310–8. 10.1159/000343320 23160634

[86] Wei Q Li YX Liu M Li X Tang H. MiR-17–5p targets TP53INP1 and regulates cell proliferation and apoptosis of cervical cancer cells. IUBMB Life. (2012) 64:697–704. 10.1002/iub.1051 22730212

[87] Xu J Li Y Wang F Wang X Cheng B Ye F. Suppressed miR-424 expression via upregulation of target gene Chk1 contributes to the progression of cervical cancer. Oncogene. (2013) 32:976–87. 10.1038/onc.2012.121 22469983

[88] Kogo R How C Chaudary N Bruce J Shi W Hill RP. The microRNA-218~Survivin axis regulates migration, invasion, and lymph node metastasis in cervical cancer. Oncotarget. (2015) 6:1090–100. 10.18632/oncotarget.2836 25473903 PMC4359219

[89] Wang F Li Y Zhou J Xu J Peng C Ye F. miR-375 is down-regulated in squamous cervical cancer and inhibits cell migration and invasion via targeting transcription factor SP1. Am J Pathol. (2011) 179:2580–8. 10.1016/j.ajpath.2011.07.037 21945323 PMC3204087

[90] Pang RT Leung CO Ye TM Liu W Chiu PC Lam KK. MicroRNA-34a suppresses invasion through downregulation of Notch1 and Jagged1 in cervical carcinoma and choriocarcinoma cells. Carcinogenesis. (2010) 31:1037–44. 10.1093/carcin/bgq066 20351093

[91] Li XR Chu HJ Lv T Wang L Kong SF Dai SZ. miR-342-3p suppresses proliferation, migration and invasion by targeting FOXM1 in human cervical cancer. FEBS Lett. (2014) 588:3298–307. 10.1016/j.febslet.2014.07.020 25066298

[92] Li MY Hu XX. Meta-analysis of microRNA expression profiling studies in human cervical cancer. Med Oncol. (2015) 32:510. 10.1007/s12032-015-0510-5 25920605

[93] Meng X Zhao Y Wang J Gao Z Geng Q Liu X. Regulatory roles of miRNA-758 and matrix extracellular phosphoglycoprotein in cervical cancer. Exp Ther Med. (2017) 14:2789–94. 10.3892/etm.2017.4887 28928798 PMC5590035

[94] Ye J Cheng XD Cheng B Cheng YF Chen XJ Lu WG. MiRNA detection in cervical exfoliated cells for missed high-grade lesions in women with LSIL/CIN1 diagnosis after colposcopy-guided biopsy. BMC Cancer. (2019) 19:112. 10.1186/s12885-019-5311-3 30700264 PMC6354336

[95] Tian Q Li Y Wang F Li Y Xu J Shen Y. MicroRNA detection in cervical exfoliated cells as a triage for human papillomavirus-positive women. J Natl Cancer Inst. (2014) 106:dju241. 10.1093/jnci/dju241 25190727 PMC4188123

[96] Kawai S Fujii T Kukimoto I Yamada H Yamamoto N Kuroda M. Identification of miRNAs in cervical mucus as a novel diagnostic marker for cervical neoplasia. Sci Rep. (2018) 8:7070. 10.1038/s41598-018-25310-1 29728572 PMC5935744

[97] Zhao S Yao D Chen J Ding N. Circulating miRNA-20a and miRNA-203 for screening lymph node metastasis in early stage cervical cancer. Genet Test Mol Biomarkers. (2013) 17:631–6. 10.1089/gtmb.2013.0085 23819812

[98] Liu P Xin F Ma CF. Clinical significance of serum miR-196a in cervical intraepithelial neoplasia and cervical cancer. Genet Mol Res. (2015) 14:17995–8002. 10.4238/2015.December.22.25 26782446

[99] Zhang Y Zhang D Wang F Xu D Guo Y Cui W. Serum miRNAs panel (miR-16–2^*^, miR-195, miR-2861, miR-497) as novel non-invasive biomarkers for detection of cervical cancer. Sci Rep. (2015) 5:17942. 10.1038/srep17942 26656154 PMC4677300

[100] Xin F Liu P Ma CF. A circulating serum miRNA panel as early detection biomarkers of cervical intraepithelial neoplasia. Eur Rev Med Pharmacol Sci. (2016) 20:4846–51. 27981553

[101] Li C Zheng X Li W Bai F Lyu J Meng QH. Serum miR-486–5p as a diagnostic marker in cervical cancer: with investigation of potential mechanisms. BMC Cancer. (2018) 18:61. 10.1186/s12885-017-3753-z 29316891 PMC5759341

[102] Wang WT Zhao YN Yan JX Weng MY Wang Y Chen YQ. Differentially expressed microRNAs in the serum of cervical squamous cell carcinoma patients before and after surgery. J Hematol Oncol. (2014) 7:6. 10.1186/1756-8722-7-6 24405714 PMC3892020

[103] You W Wang Y Zheng J. Plasma miR-127 and miR-218 Might Serve as Potential Biomarkers for Cervical Cancer. Reprod Sci. (2015) 22:1037–41. 10.1177/1933719115570902 25701838

[104] Yu J Wang Y Dong R Huang X Ding S Qiu H. Circulating microRNA-218 was reduced in cervical cancer and correlated with tumor invasion. J Cancer Res Clin Oncol. (2012) 138:671–4. 10.1007/s00432-012-1147-9 22237456 PMC11824354

[105] Jiang W Pan JJ Deng YH Liang MR Yao LH. Down-regulated serum microRNA-101 is associated with aggressive progression and poor prognosis of cervical cancer. J Gynecol Oncol. (2017) 28:e75. 10.3802/jgo.2017.28.e75 29027393 PMC5641525

[106] Tang BB Liu SY Zhan YU Wei LQ Mao XL Wang J. microRNA-218 expression and its association with the clinicopathological characteristics of patients with cervical cancer. Exp Ther Med. (2015) 10:269–74. 10.3892/etm.2015.2455 26170947 PMC4486885

[107] Lui WO Pourmand N Patterson BK Fire A. Patterns of known and novel small RNAs in human cervical cancer. Cancer Res. (2007) 67:6031–43. 10.1158/0008-5472.CAN-06-0561 17616659

[108] Lin S Gregory RI. MicroRNA biogenesis pathways in cancer. Nat Rev Cancer. (2015) 15:321–33. 10.1038/nrc3932 25998712 PMC4859809

[109] Muralidhar B Goldstein LD Ng G Winder DM Palmer RD Gooding EL. Global microRNA profiles in cervical squamous cell carcinoma depend on Drosha expression levels. J Pathol. (2007) 212:368–77. 10.1002/path.2179 17471471

[110] Pereira PM Marques JP Soares AR Carreto L Santos MA. MicroRNA expression variability in human cervical tissues. PLoS ONE. (2010) 5:e11780. 10.1371/journal.pone.0011780 20668671 PMC2909898

[111] Nahand JS Taghizadeh-Boroujeni S Karimzadeh M Borran S Pourhanifeh MH Moghoofei M. microRNAs: new prognostic, diagnostic, and therapeutic biomarkers in cervical cancer. J Cell Physiol. (2019) 234:17064–99. 10.1002/jcp.28457 30891784

[112] Berti FCB Salviano-Silva A Beckert HC de Oliveira KB Cipolla GA Malheiros D. From squamous intraepithelial lesions to cervical cancer: circulating microRNAs as potential biomarkers in cervical carcinogenesis. Biochim Biophys Acta Rev Cancer. (2019) 1872:188306. 10.1016/j.bbcan.2019.08.001 31398380

[113] Deng Y Xiong Y Liu Y. miR-376c inhibits cervical cancer cell proliferation and invasion by targeting BMI1. Int J Exp Pathol. (2016) 97:257–65. 10.1111/iep.12177 27345009 PMC4960580

[114] Shao S Wang C Wang S Zhang H Zhang Y. LncRNA STXBP5-AS1 suppressed cervical cancer progression via targeting miR-96–5p/PTEN axis. Biomed Pharmacother. (2019) 117:109082. 10.1016/j.biopha.2019.109082 31212131

[115] Natalia MA Alejandro GT Virginia TJ Alvarez-Salas LM. MARK1 is a novel target for miR-125a-5p: implications for cell migration in cervical tumor cells. Microrna. (2018) 7:54–61. 10.2174/2211536606666171024160244 29076440

[116] Navarro F Lieberman J. miR-34 and p53: new insights into a complex functional relationship. PLoS ONE. (2015) 10:e0132767. 10.1371/journal.pone.0132767 26177460 PMC4503669

[117] Wang X Meyers C Guo M Zheng ZM. Upregulation of p18Ink4c expression by oncogenic HPV E6 via p53-miR-34a pathway. Int J Cancer. (2011) 129:1362–72. 10.1002/ijc.25800 21128241 PMC3086996

[118] Martinez I Gardiner AS Board KF Monzon FA Edwards RP Khan SA. Human papillomavirus type 16 reduces the expression of microRNA-218 in cervical carcinoma cells. Oncogene. (2008) 27:2575–82. 10.1038/sj.onc.1210919 17998940 PMC2447163

[119] Liu X. Up-regulation of miR-20a by HPV16 E6 exerts growth-promoting effects by targeting PDCD6 in cervical carcinoma cells. Biomed Pharmacother. (2018) 102:996–1002. 10.1016/j.biopha.2018.03.154 29710555

[120] Cheng Y Geng L Zhao L Zuo P Wang J. Human papillomavirus E6-regulated microRNA-20b promotes invasion in cervical cancer by targeting tissue inhibitor of metalloproteinase 2. Mol Med Rep. (2017) 16:5464–70. 10.3892/mmr.2017.7231 28849054 PMC5647092

[121] Mandal P Saha SS Sen S Bhattacharya A Bhattacharya NP Bucha S. Cervical cancer subtypes harbouring integrated and/or episomal HPV16 portray distinct molecular phenotypes based on transcriptome profiling of mRNAs and miRNAs. Cell Death Discov. (2019) 5:81. 10.1038/s41420-019-0154-x 30937183 PMC6433907

[122] Qian K Pietila T Ronty M Michon F Frilander MJ Ritari J. Identification and validation of human papillomavirus encoded microRNAs. PLoS ONE. (2013) 8:e70202. 10.1371/journal.pone.0070202 23936163 PMC3728184

[123] Wu S Chen H. Anti-Condyloma acuminata mechanism of microRNAs-375 modulates HPV in cervical cancer cells via the UBE3A and IGF-1R pathway. Oncol Lett. (2018) 16:3241–7. 10.3892/ol.2018.8983 30127920 PMC6096279

[124] Shukla V Varghese VK Kabekkodu SP Mallya S Chakrabarty S Jayaram P. Enumeration of deregulated miRNAs in liquid and tissue biopsies of cervical cancer. Gynecol Oncol. (2019) 155:135–43. 10.1016/j.ygyno.2019.08.012 31434614

[125] Li H Liefke R Jiang J Kurland JV Tian W Deng P. Polycomb-like proteins link the PRC2 complex to CpG islands. Nature. (2017) 549:287–91. 10.1038/nature23881 28869966 PMC5937281

[126] Jandrig B Seitz S Hinzmann B Arnold W Micheel B Koelble K. ST18 is a breast cancer tumor suppressor gene at human chromosome 8q11.2. Oncogene. (2004) 23:9295–302. 10.1038/sj.onc.1208131 15489893

[127] Yang J Siqueira MF Behl Y Alikhani M Graves DT. The transcription factor ST18 regulates proapoptotic and proinflammatory gene expression in fibroblasts. FASEB J. (2008) 22:3956–67. 10.1096/fj.08-111013 18676404 PMC2574028

[128] Ma Q Wan G Wang S Yang W Zhang J Yao X. Serum microRNA-205 as a novel biomarker for cervical cancer patients. Cancer Cell Int. (2014) 14:81. 10.1186/s12935-014-0081-0 25788864 PMC4364049

[129] Sharma S Mandal P Sadhukhan T Roy CR Ranjan MN Chakravarty B. Bridging links between long noncoding RNA HOTAIR and HPV oncoprotein E7 in cervical cancer pathogenesis. Sci Rep. (2015) 5:11724. 10.1038/srep11724 26152361 PMC4495428

[130] Liu M Jia J Wang X Liu Y Wang C Fan R. Long non-coding RNA HOTAIR promotes cervical cancer progression through regulating BCL2 via targeting miR-143–3p. Cancer Biol Ther. (2018) 19:391–9. 10.1080/15384047.2018.1423921 29336659 PMC5915047

[131] Zhang D Zhou XH Zhang J Zhou YX Ying J Wu GQ. Propofol promotes cell apoptosis via inhibiting HOTAIR mediated mTOR pathway in cervical cancer. Biochem Biophys Res Commun. (2015) 468:561–7. 10.1016/j.bbrc.2015.10.129 26523512

[132] Lee M Kim HJ Kim SW Park SA Chun KH Cho NH. The long non-coding RNA HOTAIR increases tumour growth and invasion in cervical cancer by targeting the Notch pathway. Oncotarget. (2016) 7:44558–71. 10.18632/oncotarget.10065 27323817 PMC5190118

[133] Kim HJ Lee DW Yim GW Nam EJ Kim S Kim SW. Long non-coding RNA HOTAIR is associated with human cervical cancer progression. Int J Oncol. (2015) 46:521–30. 10.3892/ijo.2014.2758 25405331 PMC4277242

[134] Huang L Liao LM Liu AW Wu JB Cheng XL Lin JX. Overexpression of long noncoding RNA HOTAIR predicts a poor prognosis in patients with cervical cancer. Arch Gynecol Obstet. (2014) 290:717–23. 10.1007/s00404-014-3236-2 24748337

[135] Li D Feng J Wu T Wang Y Sun Y Ren J. Long intergenic noncoding RNA HOTAIR is overexpressed and regulates PTEN methylation in laryngeal squamous cell carcinoma. Am J Pathol. (2013) 182:64–70. 10.1016/j.ajpath.2012.08.042 23141928

[136] Li L Liu B Wapinski OL Tsai MC Qu K Zhang J. Targeted disruption of Hotair leads to homeotic transformation and gene derepression. Cell Rep. (2013) 5:3–12. 10.1016/j.celrep.2013.09.003 24075995 PMC4038295

[137] Ding C Cheng S Yang Z Lv Z Xiao H Du C. Long non-coding RNA HOTAIR promotes cell migration and invasion via down-regulation of RNA binding motif protein 38 in hepatocellular carcinoma cells. Int J Mol Sci. (2014) 15:4060–76. 10.3390/ijms15034060 24663081 PMC3975384

[138] Zhang H Cai K Wang J Wang X Cheng K Shi F. MiR-7, inhibited indirectly by lincRNA HOTAIR, directly inhibits SETDB1 and reverses the EMT of breast cancer stem cells by downregulating the STAT3 pathway. Stem Cells. (2014) 32:2858–68. 10.1002/stem.1795 25070049

[139] Li Q Feng Y Chao X Shi S Liang M Qiao Y. HOTAIR contributes to cell proliferation and metastasis of cervical cancer via targetting miR-23b/MAPK1 axis. Biosci Rep. (2018) 38:BSR20171563. 10.1042/BSR20171563 29335299 PMC5803494

[140] Kim SJ Park SE Lee C Lee SY Jo JH Kim JM. Alterations in promoter usage and expression levels of insulin-like growth factor-II and H19 genes in cervical carcinoma exhibiting biallelic expression of IGF-II. Biochim Biophys Acta. (2002) 1586:307–15. 10.1016/S0925-4439(01)00109-0 11997082

[141] Sun R Qin C Jiang B Fang S Pan X Peng L. Down-regulation of MALAT1 inhibits cervical cancer cell invasion and metastasis by inhibition of epithelial-mesenchymal transition. Mol Biosyst. (2016) 12:952–62. 10.1039/C5MB00685F 26798987

[142] Ling H Spizzo R Atlasi Y Nicoloso M Shimizu M Redis RS. CCAT2, a novel noncoding RNA mapping to 8q24, underlies metastatic progression and chromosomal instability in colon cancer. Genome Res. (2013) 23:1446–61. 10.1101/gr.152942.112 23796952 PMC3759721

[143] Fan MJ Zou YH He PJ Zhang S Sun XM Li CZ. Long non-coding RNA SPRY4-IT1 promotes epithelial-mesenchymal transition of cervical cancer by regulating the miR-101-3p/ZEB1 axis. Biosci Rep. (2019) 39:BSR20181339. 10.1042/BSR20181339 31092700 PMC6549091

[144] Gao J Liu L Li G Cai M Tan C Han X. LncRNA GAS5 confers the radio sensitivity of cervical cancer cells via regulating miR-106b/IER3 axis. Int J Biol Macromol. (2019) 126:994–1001. 10.1016/j.ijbiomac.2018.12.176 30579899

[145] Yang M Zhai X Xia B Wang Y Lou G. Long noncoding RNA CCHE1 promotes cervical cancer cell proliferation via upregulating PCNA. Tumour Biol. (2015) 36:7615–22. 10.1007/s13277-015-3465-4 25921283

[146] Peng W Fan H. Long noncoding RNA CCHE1 indicates a poor prognosis of hepatocellular carcinoma and promotes carcinogenesis via activation of the ERK/MAPK pathway. Biomed Pharmacother. (2016) 83:450–455. 10.1016/j.biopha.2016.06.056 27427851

[147] Zhang J Yao T Wang Y Yu J Liu Y Lin Z. Long noncoding RNA MEG3 is downregulated in cervical cancer and affects cell proliferation and apoptosis by regulating miR-21. Cancer Biol Ther. (2016) 17:104–13. 10.1080/15384047.2015.1108496 26574780 PMC4847830

[148] Xu QF Tang YX Wang X. LncRNA EBIC promoted proliferation, metastasis and cisplatin resistance of ovarian cancer cells and predicted poor survival in ovarian cancer patients. Eur Rev Med Pharmacol Sci. (2018) 22:4440–7. 10.26355/eurrev_201807_15495 30058681

[149] Sun NX Ye C Zhao Q Zhang Q Xu C Wang SB. Long noncoding RNA-EBIC promotes tumor cell invasion by binding to EZH2 and repressing E-cadherin in cervical cancer. PLoS ONE. (2014) 9:e100340. 10.1371/journal.pone.0100340 25007342 PMC4090119

[150] Guan Y Kuo WL Stilwell JL Takano H Lapuk AV Fridlyand J. Amplification of PVT1 contributes to the pathophysiology of ovarian and breast cancer. Clin Cancer Res. (2007) 13:5745–55. 10.1158/1078-0432.CCR-06-2882 17908964

[151] Barsotti AM Beckerman R Laptenko O Huppi K Caplen NJ Prives C. p53-dependent induction of PVT1 and miR-1204. J Biol Chem. (2012) 287:2509–19. 10.1074/jbc.M111.322875 22110125 PMC3268411

[152] Li MY Tang XH Fu Y Wang TJ Zhu JM. Regulatory mechanisms and clinical applications of the long non-coding RNA PVT1 in cancer treatment. Front Oncol. (2019) 9:787. 10.3389/fonc.2019.00787 31497532 PMC6712078

[153] Iden M Fye S Li K Chowdhury T Ramchandran R Rader JS. The lncRNA PVT1 contributes to the cervical cancer phenotype and associates with poor patient prognosis. PLoS ONE. (2016) 11:e0156274. 10.1371/journal.pone.0156274 27232880 PMC4883781

[154] Zhang S Zhang G Liu J. Long noncoding RNA PVT1 promotes cervical cancer progression through epigenetically silencing miR-200b. APMIS. (2016) 124:649–58. 10.1111/apm.12555 27272214

[155] Shen CJ Cheng YM Wang CL. LncRNA PVT1 epigenetically silences miR-195 and modulates EMT and chemoresistance in cervical cancer cells. J Drug Target. (2017) 25:637–44. 10.1080/1061186X.2017.1307379 28296507

[156] Yang JP Yang XJ Xiao L Wang Y. Long noncoding RNA PVT1 as a novel serum biomarker for detection of cervical cancer. Eur Rev Med Pharmacol Sci. (2016) 20:3980–6. 27775803

[157] Wang C Zou H Yang H Wang L Chu H Jiao J. Long noncoding RNA plasmacytoma variant translocation 1 gene promotes the development of cervical cancer via the NFkappaB pathway. Mol Med Rep. (2019) 20:2433–40. 10.3892/mmr.2019.10479 31322217

[158] Ma S Deng X Yang Y Zhang Q Zhou T Liu Z. The lncRNA LINC00675 regulates cell proliferation, and invasion by affecting Wnt/beta-catenin signaling in cervical cancer. Biomed Pharmacother. (2018) 108:1686–93. 10.1016/j.biopha.2018.10.011 30372871

[159] Rui X Xu Y Jiang X Ye W Huang Y Jiang J. Long non-coding RNA C5orf66-AS1 promotes cell proliferation in cervical cancer by targeting miR-637/RING1 axis. Cell Death Dis. (2018) 9:1175–228. 10.1038/s41419-018-1228-z 30518760 PMC6281646

[160] Barr JA Hayes KE Brownmiller T Harold AD Jagannathan R Lockman PR. Long non-coding RNA FAM83H-AS1 is regulated by human papillomavirus 16 E6 independently of p53 in cervical cancer cells. Sci Rep. (2019) 9:3662. 10.1038/s41598-019-40094-8 30842470 PMC6403315

[161] Shen H Wang L Xiong J Ren C Gao C Ding W. Long non-coding RNA CCAT1 promotes cervical cancer cell proliferation and invasion by regulating the miR-181a-5p/MMP14 axis. Cell Cycle. (2019) 18:1110–21. 10.1080/15384101.2019.1609829 31084453 PMC6592243

[162] Wang Q Ding J Nan G Lyu Y Ni G. LncRNA NOC2L-4.1 functions as a tumor oncogene in cervical cancer progression by regulating the miR-630/YAP1 pathway. J Cell Biochem. (2019) 120:16913–20. 10.1002/jcb.28949 31099044

[163] Qi C Xiaofeng C Dongen L Liang Y Liping X Yue H. Long non-coding RNA MACC1-AS1 promoted pancreatic carcinoma progression through activation of PAX8/NOTCH1 signaling pathway. J Exp Clin Cancer Res. (2019) 38:344. 10.1186/s13046-019-1332-7 31391063 PMC6686482

[164] Liu Q Guo X Que S Yang X Fan H Liu M. LncRNA RSU1P2 contributes to tumorigenesis by acting as a ceRNA against let-7a in cervical cancer cells. Oncotarget. (2017) 8:43768–81. 10.18632/oncotarget.10844 27487126 PMC5546439

[165] Aalijahan H Ghorbian S. Long non-coding RNAs and cervical cancer. Exp Mol Pathol. (2019) 106:7–16. 10.1016/j.yexmp.2018.11.010 30471246

[166] Chen FJ Sun M Li SQ Wu QQ Ji L Liu ZL. Upregulation of the long non-coding RNA HOTAIR promotes esophageal squamous cell carcinoma metastasis and poor prognosis. Mol Carcinog. (2013) 52:908–15. 10.1002/mc.21944 24151120

[167] Ren Y Wang YF Zhang J Wang QX Han L Mei M. Targeted design and identification of AC1NOD4Q to block activity of HOTAIR by abrogating the scaffold interaction with EZH2. Clin Epigenetics. (2019) 11:29. 10.1186/s13148-019-0624-2 30764859 PMC6376746

[168] Zhang JX Han L Bao ZS Wang YY Chen LY Yan W. HOTAIR, a cell cycle-associated long noncoding RNA and a strong predictor of survival, is preferentially expressed in classical and mesenchymal glioma. Neuro Oncol. (2013) 15:1595–603. 10.1093/neuonc/not131 24203894 PMC3829598

[169] Guo L Lu X Zheng L Liu X Hu M. Association of long non-coding RNA HOTAIR polymorphisms with cervical cancer risk in a chinese population. PLoS ONE. (2016) 11:e0160039. 10.1371/journal.pone.0160039 27467165 PMC4965140

[170] Sharma Saha S Roy CR Mondal NR Chakravarty B Chatterjee T Roy S Identification of genetic variation in the lncRNA HOTAIR associated with HPV16-related cervical cancer pathogenesis. Cell Oncol. (2016) 39:559–72. 10.1007/s13402-016-0298-0 PMC13001853 27683269

[171] Lecerf C Le BX Adriaenssens E. The long non-coding RNA H19: an active player with multiple facets to sustain the hallmarks of cancer. Cell Mol Life Sci. (2019) 76:4673–87. 10.1007/s00018-019-03240-z 31338555 PMC11105575

[172] Kallen AN Zhou XB Xu J Qiao C Ma J Yan L. The imprinted H19 lncRNA antagonizes let-7 microRNAs. Mol Cell. (2013) 52:101–12. 10.1016/j.molcel.2013.08.027 24055342 PMC3843377

[173] Matouk IJ DeGroot N Mezan S Ayesh S bu-lail R Hochberg A. The H19 non-coding RNA is essential for human tumor growth. PLoS ONE. (2007) 2:e845. 10.1371/journal.pone.0000845 17786216 PMC1959184

[174] Ou L Wang D Zhang H Yu Q Hua F. Decreased expression of miR-138–5p by lncRNA H19 in cervical cancer promotes tumor proliferation. Oncol Res. (2018) 26:401–10. 10.3727/096504017X15017209042610 28797320 PMC7844697

[175] Sun Y Ma L. New insights into long non-coding RNA MALAT1 in cancer and metastasis. Cancers. (2019) 11:E216. 10.3390/cancers11020216 30781877 PMC6406606

[176] Wilusz JE Freier SM Spector DL. 3' end processing of a long nuclear-retained noncoding RNA yields a tRNA-like cytoplasmic RNA. Cell. (2008) 135:919–32. 10.1016/j.cell.2008.10.012 19041754 PMC2722846

[177] Jiang Y Li Y Fang S Jiang B Qin C Xie P. The role of MALAT1 correlates with HPV in cervical cancer. Oncol Lett. (2014) 7:2135–41. 10.3892/ol.2014.1996 24932303 PMC4049771

[178] Lu H He Y Lin L Qi Z Ma L Li L. Long non-coding RNA MALAT1 modulates radiosensitivity of HR-HPV+ cervical cancer via sponging miR-145. Tumour Biol. (2016) 37:1683–91. 10.1007/s13277-015-3946-5 26311052

[179] Yang L Bai HS Deng Y Fan L. High MALAT1 expression predicts a poor prognosis of cervical cancer and promotes cancer cell growth and invasion. Eur Rev Med Pharmacol Sci. (2015) 19:3187–93. 26400521

[180] Guo F Li Y Liu Y Wang J Li Y Li G. Inhibition of metastasis-associated lung adenocarcinoma transcript 1 in CaSki human cervical cancer cells suppresses cell proliferation and invasion. Acta Biochim Biophys Sin. (2010) 42:224–9. 10.1093/abbs/gmq008 20213048

[181] Kasagi Y Oki E Ando K Ito S Iguchi T Sugiyama M. The expression of CCAT2, a novel long noncoding RNA transcript, and rs6983267 single-nucleotide polymorphism genotypes in colorectal cancers. Oncology. (2017) 92:48–54. 10.1159/000452143 27875818

[182] Zhou B Jing XY Wu JQ Xi HF Lu GJ. Down-regulation of long non-coding RNA LET is associated with poor prognosis in gastric cancer. Int J Clin Exp Pathol. (2014) 7:8893–8. 25674261 PMC4313956

[183] Wu L Jin L Zhang W Zhang L. Roles of long non-coding RNA CCAT2 in cervical cancer cell growth and apoptosis. Med Sci Monit. (2016) 22:875–9. 10.12659/MSM.897754 26983975 PMC4801156

[184] Khaitan D Dinger ME Mazar J Crawford J Smith MA Mattick JS. The melanoma-upregulated long noncoding RNA SPRY4-IT1 modulates apoptosis and invasion. Cancer Res. (2011) 71:3852–62. 10.1158/0008-5472.CAN-10-4460 21558391

[185] Jin J Chu Z Ma P Meng Y Yang Y. Long non-coding RNA SPRY4-IT1 promotes proliferation and invasion by acting as a ceRNA of miR-101-3p in colorectal cancer cells. Tumour Biol. (2017) 39:1010428317716250. 10.1177/1010428317716250 28720069

[186] Liu D Li Y Luo G Xiao X Tao D Wu X. LncRNA SPRY4-IT1 sponges miR-101-3p to promote proliferation and metastasis of bladder cancer cells through up-regulating EZH2. Cancer Lett. (2017) 388:281–91. 10.1016/j.canlet.2016.12.005 27998761

[187] Cao Y Liu Y Lu X Wang Y Qiao H Liu M. Upregulation of long noncoding RNA SPRY4-IT1 correlates with tumor progression and poor prognosis in cervical cancer. FEBS Open Bio. (2016) 6:954–60. 10.1002/2211-5463.12102 27642559 PMC5011494

[188] Schneider C King RM Philipson L. Genes specifically expressed at growth arrest of mammalian cells. Cell. (1988) 54:787–93. 10.1016/S0092-8674(88)91065-3 3409319

[189] Pickard MR Williams GT. The hormone response element mimic sequence of GAS5 lncRNA is sufficient to induce apoptosis in breast cancer cells. Oncotarget. (2016) 7:10104–16. 10.18632/oncotarget.7173 26862727 PMC4891107

[190] Cao S Liu W Li F Zhao W Qin C. Decreased expression of lncRNA GAS5 predicts a poor prognosis in cervical cancer. Int J Clin Exp Pathol. (2014) 7:6776–83. 25400758 PMC4230116

[191] Chen Y Wang CX Sun XX Wang C Liu TF Wang DJ. Long non-coding RNA CCHE1 overexpression predicts a poor prognosis for cervical cancer. Eur Rev Med Pharmacol Sci. (2017) 21:479–83. 28239824

[192] Zink F Magnusdottir DN Magnusson OT Walker NJ Morris TJ Sigurdsson A. Insights into imprinting from parent-of-origin phased methylomes and transcriptomes. Nat Genet. (2018) 50:1542–52. 10.1038/s41588-018-0232-7 30349119

[193] Paci P Colombo T Farina L. Computational analysis identifies a sponge interaction network between long non-coding RNAs and messenger RNAs in human breast cancer. BMC Syst Biol. (2014) 8:83. 10.1186/1752-0509-8-83 25033876 PMC4113672

[194] Gomez N Unzeta M Tipton KF Anderson MC O'Carroll AM. Determination of monoamine oxidase concentrations in rat liver by inhibitor binding. Biochem Pharmacol. (1986) 35:4467–72. 10.1016/0006-2952(86)90765-3 3790166

[195] Zhou CX Wang X Yang N Xue SK Li WC Xie PP. LncRNA LET function as a tumor suppressor in breast cancer development. Eur Rev Med Pharmacol Sci. (2018) 22:6002–7. 10.26355/eurrev_201809_15935 30280783

[196] Jiang S Wang HL Yang J. Low expression of long non-coding RNA LET inhibits carcinogenesis of cervical cancer. Int J Clin Exp Pathol. (2015) 8:806–11. 25755778 PMC4348863

[197] Zhang HD Jiang LH Sun DW Hou JC Ji ZL. CircRNA: a novel type of biomarker for cancer. Breast Cancer. (2018) 25:1–7. 10.1007/s12282-017-0793-9 28721656

[198] Zhu X Wang X Wei S Chen Y Chen Y Fan X. hsa_circ_0013958: a circular RNA and potential novel biomarker for lung adenocarcinoma. FEBS J. (2017) 284:2170–82. 10.1111/febs.14132 28685964

[199] Salzman J Gawad C Wang PL Lacayo N Brown PO. Circular RNAs are the predominant transcript isoform from hundreds of human genes in diverse cell types. PLoS ONE. (2012) 7:e30733. 10.1371/journal.pone.0030733 22319583 PMC3270023

[200] Han YN Xia SQ Zhang YY Zheng JH Li W. Circular RNAs: A novel type of biomarker and genetic tools in cancer. Oncotarget. (2017) 8:64551–63. 10.18632/oncotarget.18350 28969093 PMC5610025

[201] Ma HB Yao YN Yu JJ Chen XX Li HF. Extensive profiling of circular RNAs and the potential regulatory role of circRNA-000284 in cell proliferation and invasion of cervical cancer via sponging miR-506. Am J Transl Res. (2018) 10:592–604. 29511454 PMC5835825

[202] Liu J Wang D Long Z Liu J Li W. CircRNA8924 promotes cervical cancer cell proliferation, migration and invasion by competitively binding to MiR-518d-5p/519–5p family and modulating the expression of CBX8. Cell Physiol Biochem. (2018) 48:173–84. 10.1159/000491716 30007986

[203] Zhang J Zhao X Zhang J Zheng X Li F. Circular RNA hsa_circ_0023404 exerts an oncogenic role in cervical cancer through regulating miR-136/TFCP2/YAP pathway. Biochem Biophys Res Commun. (2018) 501:428–33. 10.1016/j.bbrc.2018.05.006 29738762

[204] Cai H Zhang P Xu M Yan L Liu N Wu X. Circular RNA hsa_circ_0000263 participates in cervical cancer development by regulating target gene of miR-150-5p. J Cell Physiol. (2019) 234:11391–400. 10.1002/jcp.27796 30569515

[205] Tian JDC Liang L. Involvement of circular RNA SMARCA5/microRNA-620 axis in the regulation of cervical cancer cell proliferation, invasion and migration. Eur Rev Med Pharmacol Sci. (2018) 22:8589–98. 10.26355/eurrev_201812_16622 30575898

[206] Abdelmohsen K Panda AC Munk R Grammatikakis I Dudekula DB De S. Identification of HuR target circular RNAs uncovers suppression of PABPN1 translation by CircPABPN1. RNA Biol. (2017) 14:361–9. 10.1080/15476286.2017.1279788 28080204 PMC5367248

[207] Zhou Y Zheng X Xu B Chen L Wang Q Deng H. Circular RNA hsa_circ_0004015 regulates the proliferation, invasion, and TKI drug resistance of non-small cell lung cancer by miR-1183/PDPK1 signaling pathway. Biochem Biophys Res Commun. (2019) 508:527–35. 10.1016/j.bbrc.2018.11.157 30509491

[208] Ding L Zhang H. Circ-ATP8A2 promotes cell proliferation and invasion as a ceRNA to target EGFR by sponging miR-433 in cervical cancer. Gene. (2019) 705:103–8. 10.1016/j.gene.2019.04.068 31029604

[209] Hu C Wang Y Li A Zhang J Xue F Zhu L. Overexpressed circ_0067934 acts as an oncogene to facilitate cervical cancer progression via the miR-545/EIF3C axis. J Cell Physiol. (2019) 234:9225–32. 10.1002/jcp.27601 30362562

[210] Mao Y Zhang L Li Y. circEIF4G2 modulates the malignant features of cervical cancer via the miR218/HOXA1 pathway. Mol Med Rep. (2019) 19:3714–22. 10.3892/mmr.2019.10032 30896864 PMC6471440

[211] Hong H Zhu H Zhao S Wang K Zhang N Tian Y. The novel circCLK3/miR-320a/FoxM1 axis promotes cervical cancer progression. Cell Death Dis. (2019) 10:950. 10.1038/s41419-019-2183-z 31831728 PMC6908646

[212] Zhao J Lee EE Kim J Yang R Chamseddin B Ni C. Transforming activity of an oncoprotein-encoding circular RNA from human papillomavirus. Nat Commun. (2019) 10:2300. 10.1038/s41467-019-10246-5 31127091 PMC6534539

[213] Guo J Chen M Ai G Mao W Li H Zhou J. Hsa_circ_0023404 enhances cervical cancer metastasis and chemoresistance through VEGFA and autophagy signaling by sponging miR-5047. Biomed Pharmacother. (2019) 115:108957. 10.1016/j.biopha.2019.108957 31082770

[214] Song T Xu A Zhang Z Gao F Zhao L Chen X. CircRNA hsa_circRNA_101996 increases cervical cancer proliferation and invasion through activating TPX2 expression by restraining miR-8075. J Cell Physiol. (2019) 234:14296–305. 10.1002/jcp.28128 30633364 PMC13483104

[215] Wang H Zhao Y Chen M Cui J. Identification of novel long non-coding and circular RNAs in human papillomavirus-mediated cervical cancer. Front Microbiol. (2017) 8:1720. 10.3389/fmicb.2017.01720 28970820 PMC5609541

